# Characterization of Electronic Stress-Induced Changes in Multilayer MoS_2_

**DOI:** 10.1021/acsaelm.6c00080

**Published:** 2026-04-06

**Authors:** R. Colby Evans, Riccardo Torsi, Pavel Kabos, Jason Holm, Jason P. Killgore, Paul Owiredu, Gurpreet Singh, Jerzy T. Sadowski, Angela R. Hight Walker, Elisabeth Mansfield

**Affiliations:** † 535691 National Institute of Standards and Technology, Applied Chemicals and Materials Division, Boulder, Colorado 80305, United States; ‡ 10833 National Institute of Standards and Technology, Quantum Measurement Division, Gaithersburg, Maryland 20899, United States; § National Institute of Standards and Technology, Applied Physics Division, Boulder, Colorado 80305, United States; ∥ Department of Mechanical and Nuclear Engineering, 5308 Kansas State University, Manhattan, Kansas 66506, United States; ⊥ Center for Functional Nanomaterials, 8099 Brookhaven National Laboratory, Upton, New York 11973, United States

**Keywords:** MoS_2_, transition metal dichalcogenide, electronic stress, morphological changes, multimodal imaging

## Abstract

Transition metal dichalcogenides like molybdenum disulfide (MoS_2_) are compelling for next-generation electronic devices. In this work, we investigate the impact of electronic stress on MoS_2_ to illustrate that observational and phenomenological information on multiple devices can be useful to describe changes in the device, and caution against the rationalization of paltry results as representative or correlative to device behavior. Here, we stress MoS_2_ by applying a sustained 20 V DC bias to study the material’s response. Post-stress electronic characterization revealed nonuniform shifts in current–voltage (I–V) behavior alongside microscale changes. Complementary mechanical, spectroscopic, and scanning microwave impedance measurements showed that stress-induced features locally modulate stiffness, surface potential, Raman intensity, and charge carrier density. We correlated I–V behavior with morphological features (wrinkles, tears, folds, height) and device-level geometry (MoS_2_ overlap with electrodes, channel area, contact length) on 50 test structures across five chips to move beyond anecdotal conclusions. We found no universal correlations before DC stress. However, device-level geometry was correlated with I–V behavior after DC stress, suggesting that electrode contacts play a more dominant role than morphology in determining performance. Delamination and thinning induced by DC stress led to localized reductions in charge carrier density within the affected regions. Further, delamination and thinning appear to map to I–V device performance in a few samples, but the correlation is lost when a larger sample size is considered. This suggests significant sample-to-sample variability in surface electronic states of the test structures. We also discuss how environmental factors introduced during fabrication may contribute to the observed heterogeneous device response. Progress will require high-resolution, multimodal analysis across many samples constructed under controlled, clean conditions. By building data sets that capture variability, we can better identify the true drivers of performance.

## Graphical abstract

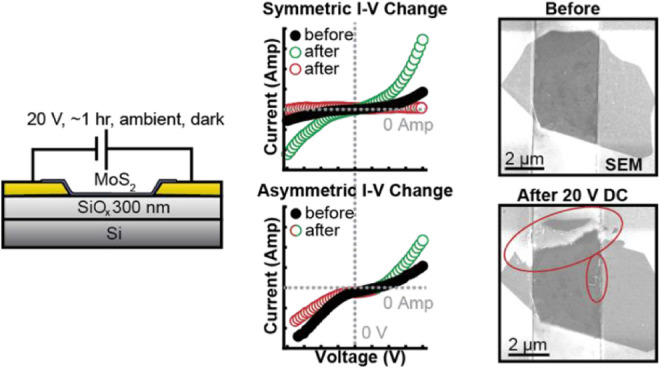

## Introduction

Next-generation computational needs require advancements in device engineering, including transistor miniaturization.^1–3^ Subnanometer-thick materials like two-dimensional transition metal dichalcogenides (TMDs) are considered strong candidates for such miniaturization.^3–6^ Among them, molybdenum disulfide (MoS_2_) possesses a sufficiently large bandgap, favorable electrostatics, and is mechanically flexible, making it suitable for field-effect transistors, flexible electronics, and optoelectronic devices.^1,2,7,8^ However, electronic stress, whether from high current densities or prolonged biasing, can drive irreversible changes, including atomic-scale defect formation, altered morphology, and spatial variations in conductivity.^9–12^ Additionally, environmental factors and contamination have been shown to play a decisive role in the performance and stability of 2D devices,^13,14^ often complicating attempts to draw structure–property correlations.

Analyzing materials after device operation and stress is essential to understand how material changes impact key performance metrics. Few studies report the impact of electronic stress on TMDs, despite their importance and the extensive literature on these materials. In 2020, Shrivastava and coworkers showed that localized high-conductance regions and amorphization of monolayer MoS_2_ led to device failure under combined gate bias and source-drain bias, with no apparent correlation between the initial current–voltage (I–V) response and degradation.^10^ They speculated that ballistic energy from electrons traveling through the channel is sufficient to reorganize the lattice from crystalline to amorphous using Raman and photoluminescence spectroscopy. Pezoldt and coworkers reported similar Raman signals in multilayer exfoliated MoS_2_ under gate-biased conditions, which demonstrates that this mechanism is not a layer-dependent property.^12^ Shrivastava and coworkers later showed that long-term electrical stress negatively shifts threshold voltage, increases channel conductance, and reduces thermal transport of MoS_2_ transistors due to a weakening of Mo–S bonds.^11^ We recently demonstrated that electronic stress in monolayer MoS_2_ leads to the expansion of nanoscale defects.^9^ A clear relation between applied stress voltage, time under stress, and material damage was observed, emphasizing the need to combine structural and electronic measurements to understand degradation and morphology changes.

Apparent device-to-device variability in MoS_2_ I–V behavior is rarely due to a single cause. Polymer residues from processing, adsorbents, contact quality, and measurement protocols each produce effects that can mimic, mask, or amplify changes.^14–16^ Without deliberate control and explicit reporting of these factors, small-N studies are prone to misinterpretation. Shrivastava et al., Pezoldt et al., and our prior work relied on polymer-based transfer methods, and, like our work, the investigation by Pezoldt et al. was conducted under ambient conditions. Notably, Shrivastava et al. reported similar morphological changes in both contact potential difference and topography, despite using carefully controlled inert environments. Ambient exposure to oxygen and water leads to adsorbate-induced charge trapping, threshold voltage shifts, and reduced on-current, especially in unpassivated MoS_2_ transistors. For example, Park et al. demonstrated that exposure to O_2_ shifts the threshold voltage in the positive direction and reduces carrier concentration, while passivated devices are much less affected.^17^ Long-term exposure studies report gradual oxidation (formation of MoO_3_ or oxidized sulfur species) that degrades electrical conductivity and mechanical properties.^18,19^ Water molecules in the ambient environment can physisorb on MoS_2_, acting as molecular gate dopants or trap states, which modify carrier mobility and subthreshold behavior.^20,21^

Establishing a direct link between morphological changes and charge carrier behavior is necessary to determine their impact on device performance. To date, studies have not explored how different electronic stress-induced material changes directly influence charge carriers and often do not consider the impact of processing, test approach, and environmental conditions on the results. In this work, we begin to address this gap by employing a suite of characterization techniques—scanning electron microscopy (SEM), atomic force microscopy (AFM), scanning Kelvin probe microscopy (SKPM), Raman spectroscopy, and mechanical measurements—to identify morphological changes resulting from electronic stress. Scanning microwave impedance microscopy (sMIM) is then used to map charge carrier density after electronic stress. This low-throughput multimodal imaging approach was applied to a select set of MoS_2_-based test structures, based on their I–V behavior after DC stress, to serve as a starting point in understanding how morphological changes impact charge carriers and how ambient process and test conditions influence data interpretation.

Comparing I–V metrics with morphological features across 50 test structures before and 28 test structures after DC bias was insufficient to determine a link. Given the limited sample size, causality between morphological changes and I–V metrics cannot yet be definitively established. In fact, one MoS_2_ flake showed no detectable changes in morphology despite a significant drop in I–V response. We caution against rationalizing generalized conclusions on structure–property relationships from small-sample-size studies. Further, environmental contamination in device processing could also play a role in heterogeneous I–V behavior; it has been established that clean transfer and fabrication of graphene-based devices drastically reduce the variation in performance.^14,22^ Herein, we demonstrate that the same care is needed for MoS_2_. Despite this heterogeneous behavior, two distinct topological changes were identified—delamination and material thinning—that have locally reduced charge carrier density. We highlight the need for new measurement techniques capable of detecting a broader range of material changes, as well as large-scale reliability studies to fully explain the mechanisms by which TMDs, like MoS_2_, change under electronic stress. We discuss the implications of testing MoS_2_-based devices in ambient conditions and encourage future studies to take seriously the impact of both sample number and sample cleanliness on results.

## Results

### Material Growth and Fabrication of Test Structures

Chemical vapor deposition (Figure 1a) was used to grow multilayer MoS_2_ (Figure 1b) on SiO_2_/Si wafers. Established wet PMMA transfer methods were used to transfer as-grown MoS_2_ onto electrodes.^23,24^ Figure 1c shows a representative top-down SEM image of the test structures fabricated for this study. Herein, the source and drain will be depicted on the left and right, respectively. Only electrode pairs bridged by a single flake were considered in this study. Figure 1d shows a side-on view cartoon of the electronic test structures. See Methods, SI Synthesis Discussion, and Figures S1–S3 for more information on synthesis and characterization.

**Figure 1:**
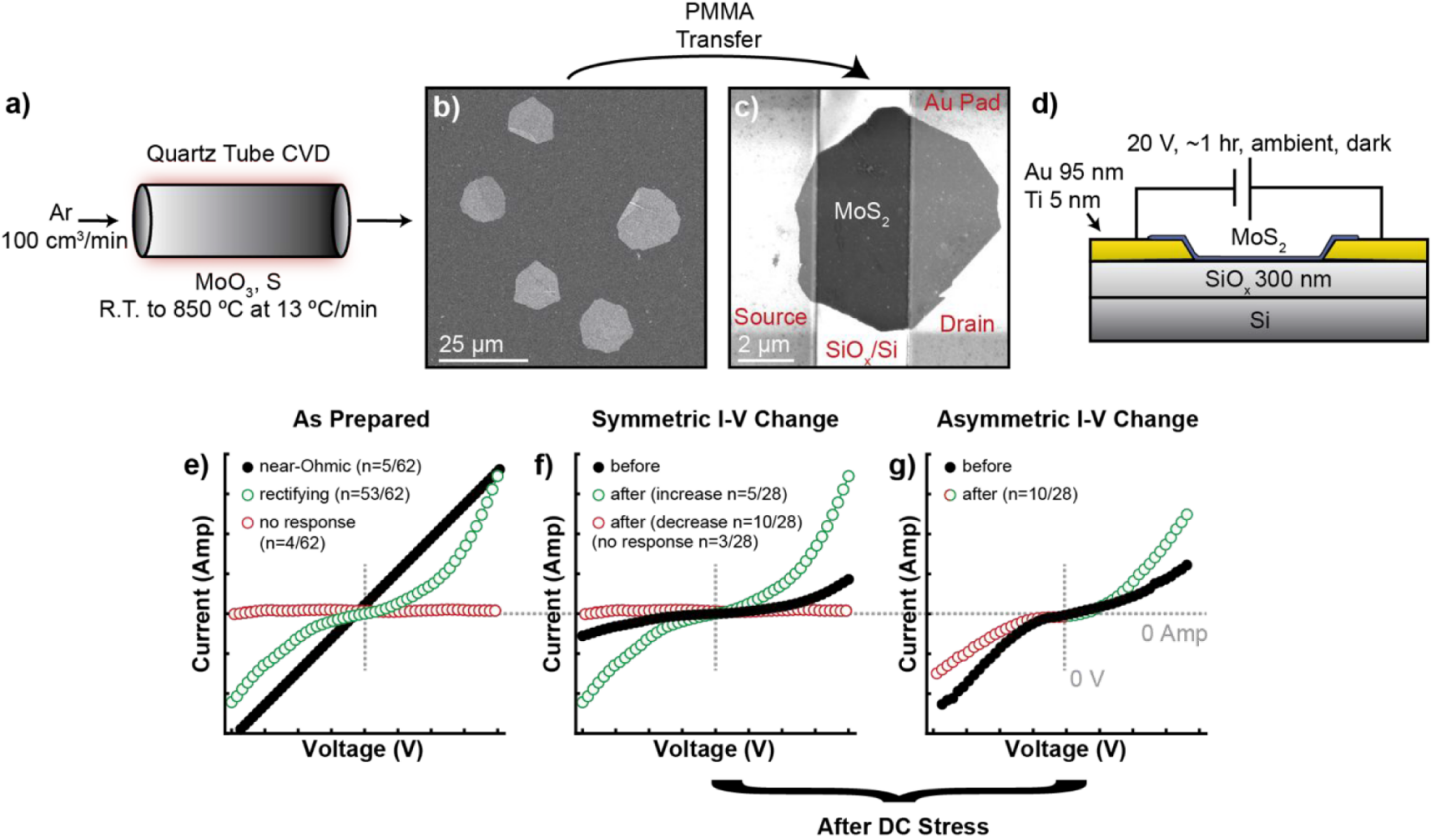
Synthesis, device fabrication, and schematic illustration of different I–V behaviors as-prepared and after DC stress. a) Scheme of the MoS_2_ synthesis using chemical vapor deposition. b) SEM image of the resulting growth on SiO*_x_*/Si. c) SEM image of a test structure. MoS_2_ is stamped across two Ti/Au current collectors on a Si wafer. d) Illustration of test structure architecture (not to scale). e) Ohmic, diode, and open circuit traces are measured on as-prepared devices. After DC stress, current can symmetrically (f) increase or decrease. g) Some flakes have polarity-dependent changes in I–V behavior, asymmetrically increasing or decreasing after DC stress. (e–g) are schematic illustrations of I–V behavior (symmetry and current magnitude).

### Impact of DC Electronic Stress on I–V Behavior

Linear current–voltage (I–V) sweeps from −20 to 20 V were done on 62 test structures across five chips in a two-terminal configuration. Figure 1e schematically illustrates the heterogeneous I–V responses. Behaviors included near-Ohmic (5/62), rectifying (53/62), and open circuit (4/62). The near-Ohmic response was concentrated (4/5) on a single chip, unlike the open circuit response and rectifying response, which were distributed across all chips. We focus on test structures that display rectifying behaviors for this study. Twenty-eight test structures were subjected to DC electronic stress, which impacted current responses and the morphology of MoS_2_ flakes to varying degrees. The maximum current (i_max_) at any potential can change in three distinct ways after DC stress: (1) symmetrically increase or decrease (15/28), (2) asymmetrically increase and decrease (10/28), and (3) open circuit (3/28). Figure 1f and g illustrate scenarios (1,2) and (3), respectively. Changes in conductivity, and subsequently i_max_, can be caused by an array of factors, including changes in dopant profile, crystallinity, layer number, stress/strain, and contact with current collectors.^25–29^ The observed asymmetry and negative differential resistance-like features may arise from nonideal Schottky contacts rather than ideal diode behavior. A multimodal microscopy approach was applied to one flake from each of the three categories to identify the types of structural changes that occur and to assess how those changes may influence performance.

#### Flake 1, Symmetric Increase in i_max_

Current–voltage, SEM, AFM, and SKPM measurements before and after DC electronic stress, as well as mechanical measurements after stress, are shown in Figure 2. Figure 2a shows that the i_max_ increases for both positive and negative polarities by 2 orders of magnitude—the flake appears to be more electrically conductive after DC electronic stress. This flake was chosen for multimodal analysis because it demonstrated one of the largest increases in i_max_ of all flakes in this category. Forward bias onset voltage (V_f_) and reverse bias onset voltage (V_r_) both shift toward 0. Applying potentials more negative than V_r_ causes the device to enter a region of reduced conductivity. The clear change in I–V characteristics suggests a change in the material, the electrode contacts, or both. SEM of Flake 1 after DC stress shows: growth of a semicircular feature from the drain into the channel, new blister-like features over the drain electrode, and bump-like features over the source (Figure 2b,c). No change in the electrode itself is observed. Topographic and nanomechanical measurements distinguish the semicircular feature on Flake 1 as delamination. Figure 2d,e shows height maps and force–distance curves collected at two locations per flake: the basal plane (circles) and regions of morphological change (squares). A line profile across the morphological feature shows an 8.7 ± 0.2 nm height increase, with 1.7 ± 0.2 nm attributed to an interior flake visible in the amplitude error map (Figure S4). Force–distance analysis reveals reduced stiffness in the region of morphological change (0.73 ± 0.01 N/m) compared to the basal plane (0.98 ± 0.03 N/m). The combined increase in height and compliance indicates delamination, though whether this occurs between MoS_2_ layers, the substrate, or both remains unclear from nanomechanical data alone.

**Figure 2:**
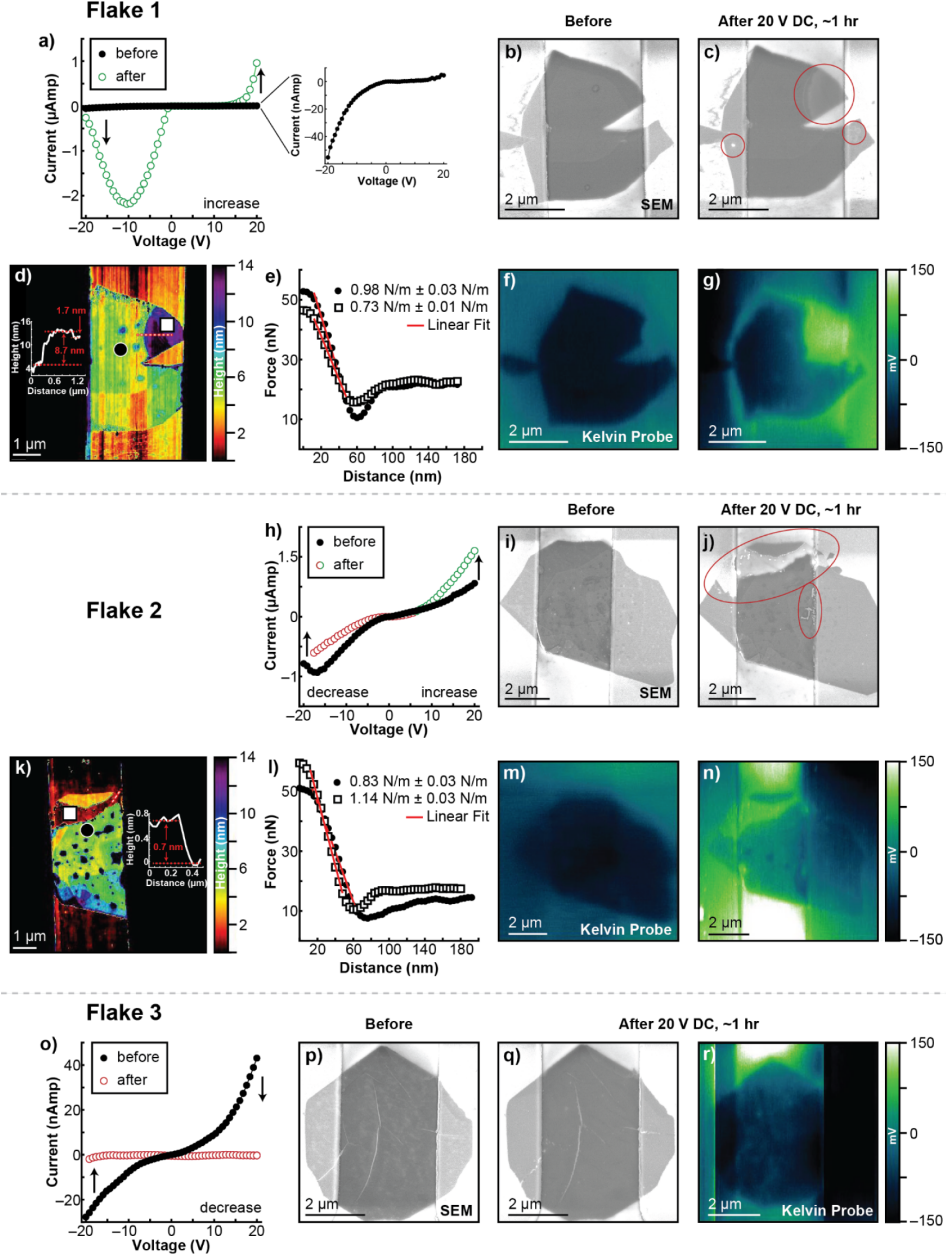
I–V and morphological characterization of Flake 1 before and after DC electronic stress. (a,h,o) Current responses before (solid circle) and after (open circle) DC bias. SEM images before (b,i,p) and after (c,j,q) 20 V DC bias for ∼1 h in ambient conditions. (d,k) Height map contrast optimized for within the channel. Insets are line profiles taken along the red and white dashed lines. (e,l) Force–distance plots for the areas indicated by the white circle symbol with black fill (on basal plane) and black square symbol with white fill (on areas of morphological change). The R^2^ of all linear fits are ≥0.98. CPD from Kelvin probe before (f,m) and after (g,n,r) DC electronic stress, respectively. CPD was normalized to the average of the electrode signal to compare MoS_2_ scanned with different cantilevers.

SKPM was used to measure the contact potential difference (CPD) before and after electronic stress. Figure 2f,g presents spatially correlated CPD maps, where delamination and blisters appear as high-CPD regions that were absent prior to stress. Additionally, the basal plane CPD increases by 21.3 ± 15 mV, consistent with increased strain.^30–32^

#### Flake 2, Asymmetric Increase in i_max_

Unlike Flake 1, Flake 2 experiences an asymmetric change in I–V characteristics. Figure 2h shows that i_max_ increases at positive potentials and decreases at negative potentials after DC electronic stress. An increase in forward bias current and a decrease in reverse bias current is a potentially favorable outcome at first glance, but any unpredictable deviation from initial device performance is unfavorable from a quality control standpoint. SEM imaging of Flake 2 shows three areas of morphological change after electronic stress (Figure 2i,j): one bridges the electrode gap, while the others are localized to the drain electrode. The large defect that spans the channel widens from ∼100 nm at the drain to ∼1 μm at the source, apparently bound by the step edge from the intraflake fold (SI Figure S4). Importantly, Flake 2 did not short despite this feature reaching both electrodes; this defect is not metallic in nature. The height map (Figure 2k) indicates the large feature spanning the electrode channel is thinner than the surrounding flake. A line profile from the thinned region to the substrate shows a height of 0.7 ± 0.2 nm—approximately a monolayer—though thickness varies across the channel. The thinned area is stiffer (1.14 ± 0.03 N/m) than the basal plane (0.83 ± 0.03 N/m); the underlying SiO_*x*_ substrate contributes more to the mechanical signal at the thinned region. Henceforth, the areas of morphological change on Flake 1 and Flake 2 will be discussed as delamination and thinning, respectively.

The thinned region has a CPD higher than that of the rest of the flake (Figure 2m,n). Further, three low-CPD regions appear post-stress, implying the formation of more strongly n-doped regions.^31^ Interpretation of CPD in the thinned area is more ambiguous than for delamination. It is possible that a chemical or crystallographic change has occurred in the region of thinning, not present in the delamination in flake 1. Higher CPD could result from the area being reduced to a monolayer^33^ or from the loss of crystallinity.^11^ Below, we discuss spatially resolved confocal Raman data that provide additional differentiation between morphological changes in flakes 1 and 2.

#### Flake 3, Loss of Current Response

Comparing the I–V trace before and after electronic stress (Figure 2o) shows a transition from rectifying behavior to open circuit. Morphological change in Flake 3 is minimal (some small surface features disappear, and a central wrinkle flattens) despite the shift in electronic behavior. While no prominent topographic changes are detected in SEM or AFM (Figure 2p,q and SI S4), CPD becomes more heterogeneous (Figure 2r) and drops by 27.6 ± 23 mV. Larger deviation in CPD in the unstressed state may indicate a higher intrinsic disorder (heterogeneous distribution of midgap states) compared to Flakes 1 and 2 (SI Figure S4).^32^ Taken altogether, results for Flake 3 suggest I–V degradation comes from either changes to the electronic structure of the material or a disruption of MoS_2_/electrodes contact, rather than morphological changes. The presence of visible changes in the material is not an indicator for the reduction in current response.

Alternatively, the changes in I–V behavior and CPD may arise from the adsorption of surface contaminants. In Flake 1, the shift in onset potentials could be interpreted as a reduction peak at negative bias and the onset of oxidation at positive bias from species that were not present in the “as-prepared” flake prior to DC stress. Flake 2, in contrast, showed a weak reduction feature before stress that disappeared afterward. These distinct responses in flakes subjected to the same conditions suggest either strong differences in preferential adsorption and resulting surface chemistry, wide variation in environmental contamination not limited to nominally equivalent PMMA residue from transfer, or that the variations in electrical behavior originate from another mechanism altogether. If indeed these current signals are due to redox rather than a change in electronic behavior, the most likely culprits are water oxidation and O_2_ reduction; the features cannot be fully explained as desorption of surface species because they persist over time and multiple voltage cycles.

### Raman Spectra of Flake 1 and Flake 2

Comparison of Raman spectra from delamination and thinning within the basal plane of Flake 1 and Flake 2 reveals differences in strain and interlayer coupling (Figure 3). Figure 3a,c shows a log intensity plot of the A_1g_ peak area. The MoS_2_ signal strength varies across the two flakes. The signal is weakest within areas of morphological change (delamination and thinning) and most intense over the electrodes. Increased signal at the electrode is likely due to plasmon enhancement.^34,35^ Single spectra were extracted from the maps to compare delamination and thinning to the basal planes of each respective flake (Figure 3b,d). There are several possibilities for the differences in signal within delamination and thinning: change in interlayer coupling,^36^ change in strain,^25,37^ and/or loss of crystallinity.^38,39^ First, we will examine Flake 1. The lack of interlayer shear and breathing modes from 10 cm^–1^ to 30 cm^–1^ in Figure 3b within spot 1 (delamination) compared to spot 2 (seven-layer (7L) and six-layer (6L) modes, basal plane) indicates the loss of coupling between layers. Notably, despite the loss of interlayer modes, the peak spacing between the A_1g_ and E^1^_2g_ modes is only weakly affected (Table 1). Instead, the E^1^_2g_ blueshifts relative to the basal plane while the A_1g_ peak remains stationary, consistent with increased compressive strain expected in buckling or delamination processes.^40^ The loss of interlayer modes provides evidence that delamination is occurring between MoS_2_ layers to some degree, rather than uniform delamination from the substrate. The signal from thinning on Flake 2 is weaker than the delamination. No strong conclusions can be drawn from the low-frequency range regarding interlayer coupling because of low signal-to-noise. Further, interpretation of A_1g_ and E^1^_2g_ peak shifts is convoluted by the loss of material thickness, which is known to impact peak position and separation.^41^ The overall loss of signal in this region may indicate a loss of crystallinity, which would be consistent with amorphization seen in other electronic stress studies.^10,12^ There are no clear Raman peaks for oxidation products or C-based contamination (Figure S5).^12,42^ All peaks correspond to known MoS_2_ modes.^43–45^

**Table 1:** Raman Peak Positions and Full Width Half Maximum for Each Spot on Flakes 1 and 2 from Figure 3

	Spot 1		Spot 2		Spot 3	
	E_2g_	A_1g_	E_2g_	A_1g_	E_2g_	A_1g_
Peak position (cm^–1^) Flake 1	384.66 ± 0.09	409.50 ± 0.03	384.21 ± 0.04	409.52 ± 0.13	-	-
fwhm (cm^–1^) Flake 1	2.18 ± 0.32	3.14 ± 0.10	1.83 ± 0.15	3.01 ± 0.04	-	-
Peak position (cm^–1^) Flake 2	385.77 ± 0.09	410.53 ± 0.07	385.28 ± 0.04	410.36 ± 0.03	385.45 ± 0.03	410.03 ± 0.02
fwhm (cm^–1^) Flake 2	2.35 ± 0.40	3.79 ± 0.26	2.84 ± 0.19	4.68 ± 0.10	2.36 ± 0.14	4.16 ± 0.08

**Figure 3:**
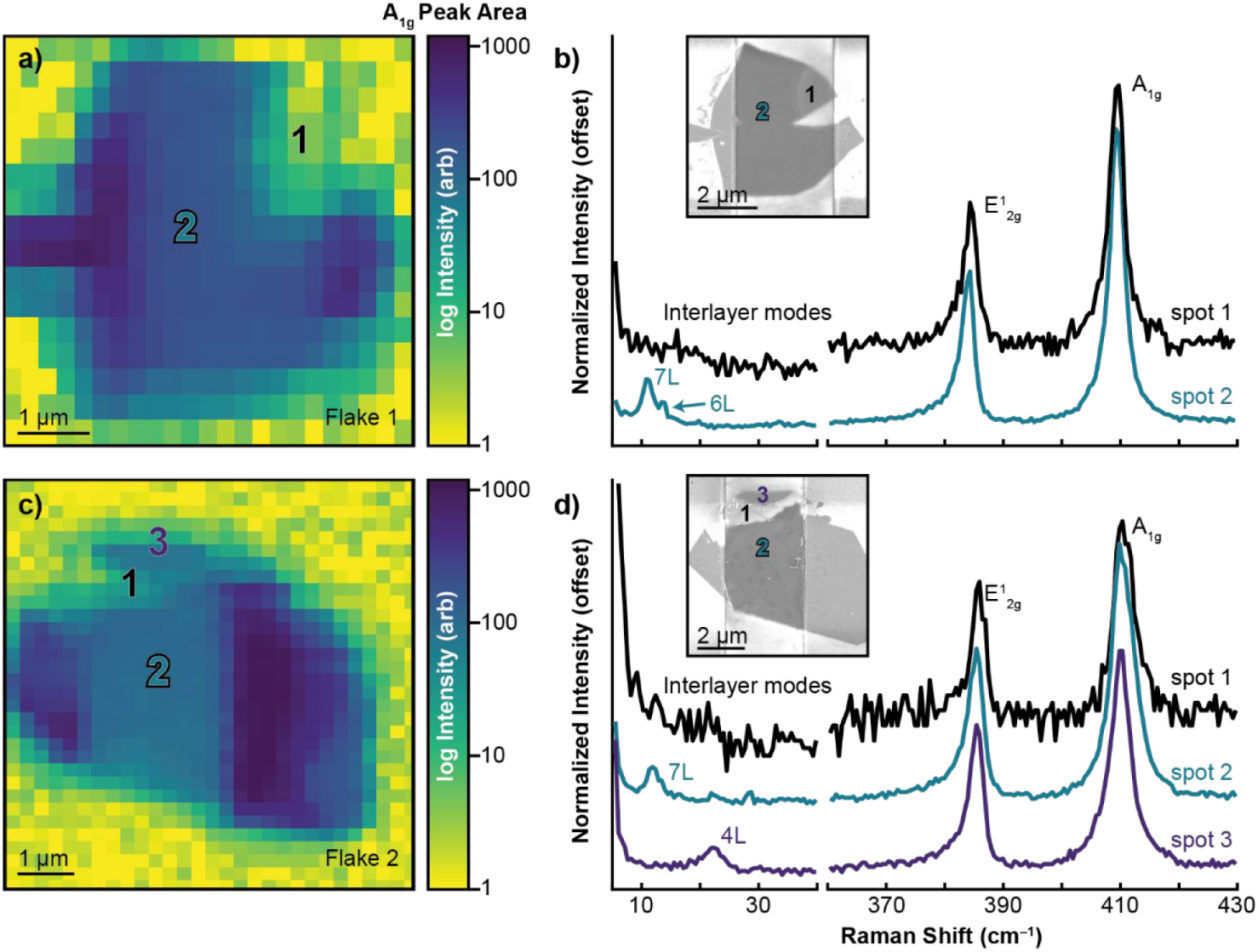
Confocal Raman spectroscopy on Flakes 1 and 2. (a,c) Confocal Raman log intensity maps of the A_1g_ peak area for Flakes 1 and 2, respectively. Numbers indicate spots corresponding to spectra plotted in b and d. (b,d) Raman spectra for spots indicated in a and c for Flakes 1 and 2, respectively. Insets are SEM images, again numerically labeled according to the plotted spectra, to show finer resolution. The excitation wavelength for all Raman experiments was 532 nm.

### Exploring the Influence of Morphological Features and Device-Level Geometry on Heterogeneous I–V Behavior

It is unclear what role morphological features (wrinkles, folds, tears) or device-level geometry (areal overlap of material on electrodes, channel area) have in explaining the heterogeneous I–V behavior before or after DC stress (Figure 1). Here, we use forward voltage onset (V_f_), reverse voltage onset (V_r_), the maximum current in the forward direction (i_f_), and the maximum current in the reverse direction (i_r_) as metrics to correlate with morphological features and device-level geometry. Figure 4 shows a top-down SEM image of a representative test structure and an illustration of a voltammogram with relevant morphological, device-level, and I–V metrics labeled. Table 2 shows the Pearson’s correlation coefficients for the resulting 40 correlations between I–V characteristics and device-level geometry (for 50/53 of the rectifying devices), including SKPM results (23/53 devices). Weak and strong correlations are highlighted in orange and green, respectively. At first glance, it appears that CPD and V_f_ are correlated—a promising sign that SKPM could be a screening tool to find poor-performing devices. However, this correlation can be explained by chip-dependent behavior (Figure S6), which convolutes interpretation. In fact, all of the correlations can be explained by chip-dependent behavior except for height. The weak correlation of current with height is not surprising; increasing layers in few-layer MoS_2_ results in more current.^46,47^ Table 3 shows an additional 40 correlations, now between the percent change (%Δ) in I–V metrics after DC stress against device geometry (20/28 of the stressed devices) or SKPM results. While correlations in CPD and height are convoluted by low sample size and chip-dependent behavior, the device-level geometry metrics become either weakly or strongly correlated with at least one %ΔI–V metric. This implies that the interaction between the electrode and MoS_2_ plays an important role in how the device changes with stress. The quality of semiconductor-metal contact is known to impact device performance and remains an active area of research.^48^

**Table 2:**
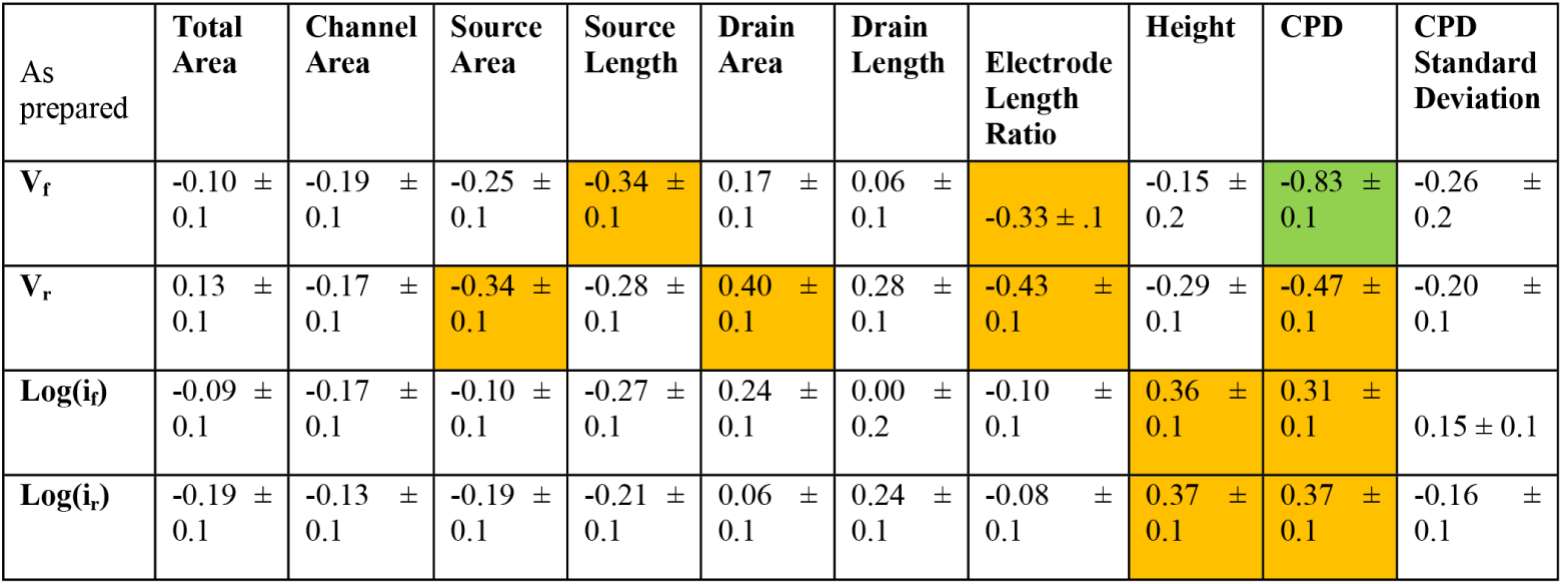
Pearson’s Correlation Coefficients Between I–V Parameters, Device Geometry, and CPD (Normalized to Au) from SKPM of As Prepared Device^a^

a Weak Correlations are Colored Yellow (> 0.30 ± 0.1 < 0.6 ± 0.1), and Strong Correlations are Colored Green (>0.60 ± 0.1).

**Table 3:**
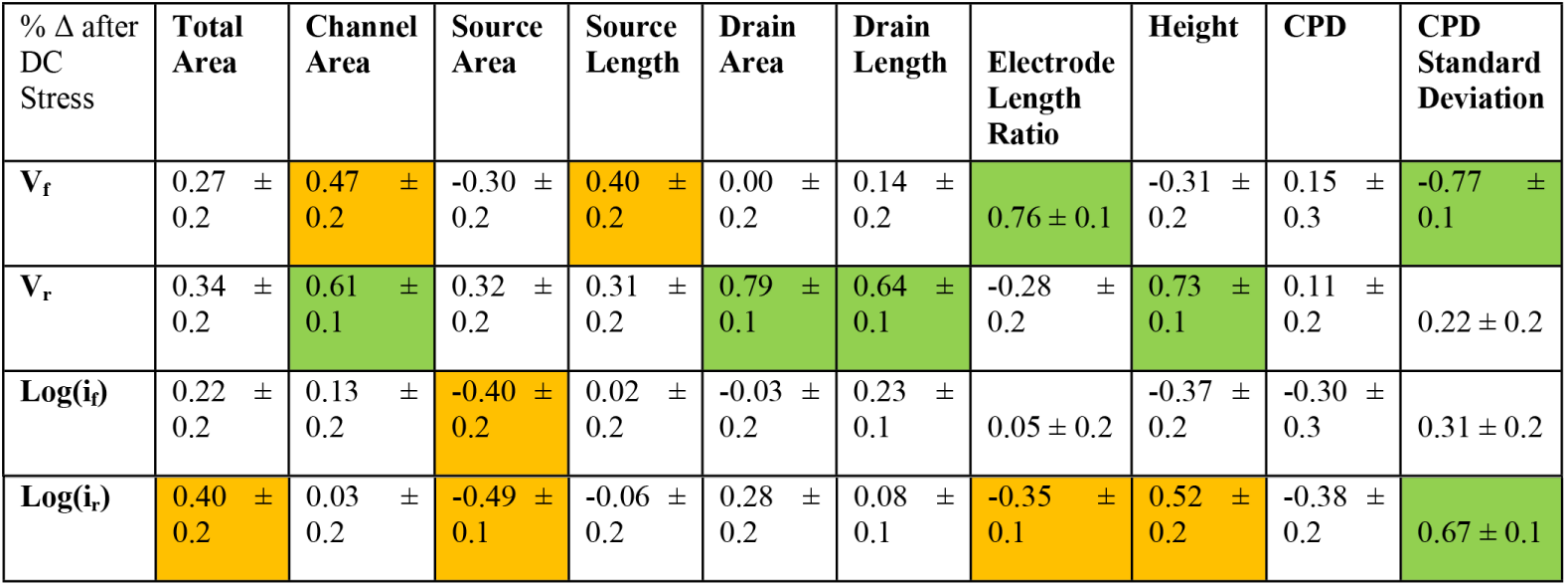
Pearson’s Correlation Coefficients Between I–V Parameters, Device Geometry, and CPD (Normalized to Au) from SKPM of as Devices after DC Electronic Stress^a^

a Weak Correlations are Colored Orange (> 0.30 ± 0.1 < 0.6 ± 0.1), and Strong Correlations are Colored Green (>0.60 ± 0.1).

**Figure 4:**
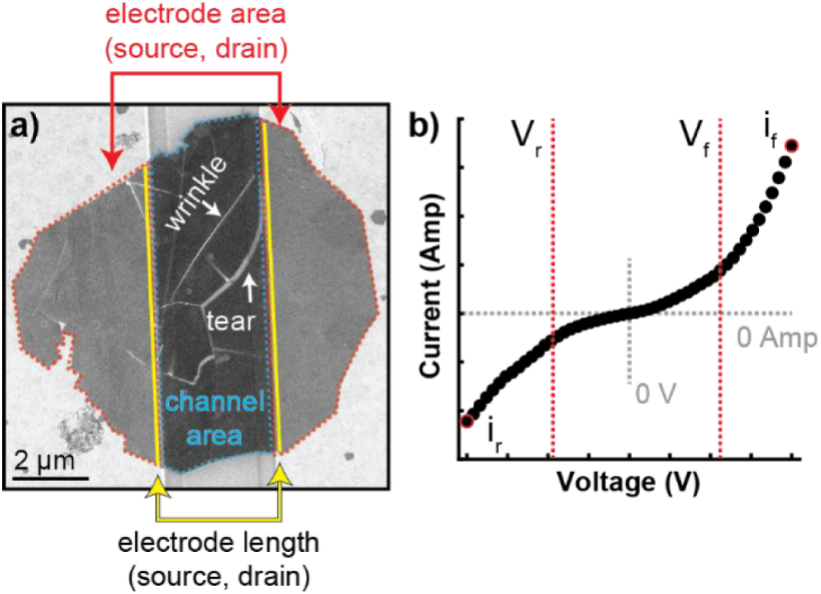
Example of device and voltammogram labeled with parameters for correlation. (a) SEM image of an MoS_2_ flake between Au electrodes. The red and blue dashed lines outline the overlap between MoS_2_ and the electrodes, and the area of MoS_2_ inside the channel, respectively. This flake has one wrinkle and one tear each indicated by white arrows. The length of contact at the interface of MoS_2_ is defined as “electrode length” and is shown as a solid yellow line. b) Illustration of a typical rectifying voltammogram labeled with the forward voltage onset (V_f_), reverse voltage onset (V_r_), maximum current in the forward direction (i_f_), and maximum current in the reverse direction (i_r_).

The impact of morphological features on I–V and %ΔI–V metrics was evaluated by comparing histograms of flakes with and without each feature (wrinkle/no wrinkle, tear/no tear, fold/no fold, and fold and wrinkle/no fold or wrinkle). In all cases, the populations overlapped— there is no distinct difference in performance between flakes with or without (flat basal plane) visible morphological features (Figures S7-S10). We conclude that morphological features do not play a role in I–V performance before or after DC electronic stress for devices fabricated in this study. Further, there is no apparent correlation between morphology and Ohmic, diode, open-circuit I–V behavior, or resulting symmetric/asymmetric changes after DC stress, as shown in Figure 1e,f,g.

The 50+ devices studied here were too small of a sample size to explain correlation or link cause to the heterogeneous I–V behavior. However, the increased strength of correlation for device-level geometry with I–V metrics (Table 3) after DC stress implies that contact with the current collectors dominates over morphological features for device performance. Environmental contamination of the gold electrodes could be a source of variation in the MoS_2_-electrode contact. Though we chose gold specifically because it is known to be relatively unreactive with TMDs,^49,50^ gold is known to contaminate in air.^51^ Further, Turetta et al. showed that surface contamination of gold is not clearly uniform in composition or geometry,^51^ which would introduce intrinsic variability into contact between the electrodes and MoS_2_ in any ambient study, including this one. Many additional devices with high-quality contact to electrodes should be evaluated before conclusions can be drawn about the impact of morphological features on I–V metrics.

### Impact of Electronic Stress on Charge Carrier Density

Based on the above statistical analysis after DC stress, features that correspond to delamination and material thinning do not correlate with any of the three I–V changes (symmetric, asymmetric, or open circuit). Therefore, conventional electron imaging, scanning probe techniques, and linear I–V sweeps are insufficient screening tools to predict how electronic stress will impact a device or connect I–V trends to morphological changes in a small-sample-size experiment. Direct, spatially resolved measurements of material conductivity are needed to rationalize how morphological changes impact the electronic properties of the material after electronic stress.

Here, sMIM was used to evaluate how electronic stress affects charge carrier density in MoS_2_. A reflected, demodulated microwave signal measures the real (resistive) and imaginary (capacitive) properties of the sample under the cantilever tip during a contact-mode scan.^52,53^ We focus on the derivative capacitance signal (dC/dV amplitude) as a proxy for local conductivity (Figure 5). High contrast corresponds to higher carrier density; low contrast indicates depletion (i.e., local conductivity).^54,55^ We stress that DC tip bias refers to the bias between the cantilever tip and the MoS_2_—not across the source and drain.

**Figure 5:**
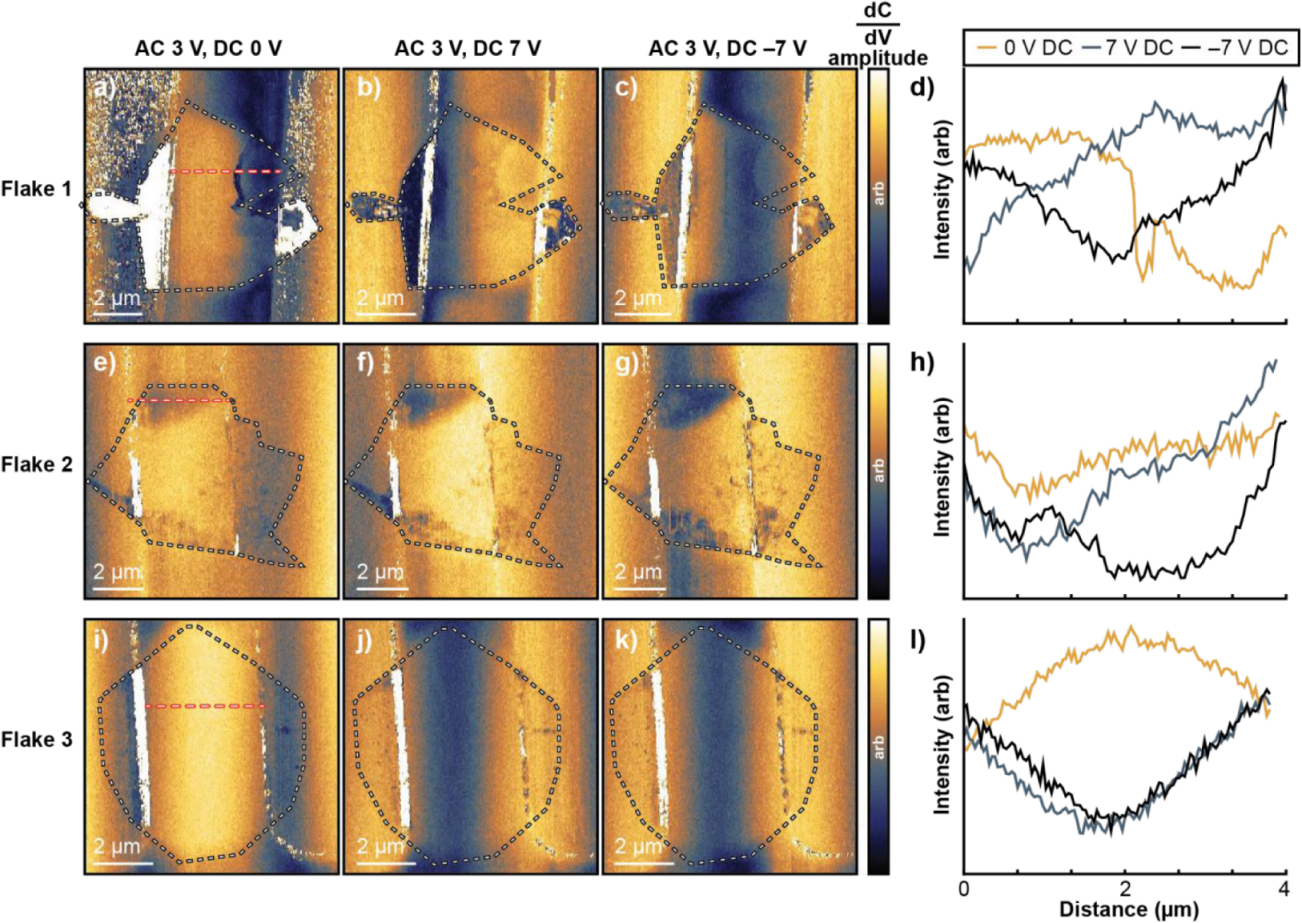
Impact of defects on charge carrier density. Differential capacitance amplitude plots from sMIM of Flakes 1, 2, and 3 under no DC tip bias (a,e,i), 7 V DC tip bias (b,f,j), and −7 V DC tip bias (c,g,k). All measurements were taken with a 3 V AC driving voltage. Black and white dashed lines are an overlay of flake shape from SEM. Red and white dashed lines are areas where line profiles were extracted under no DC tip bias (yellow), 7 V DC tip bias (blue), and −7 V DC tip bias (black) for Flake 1 (d), Flake 2 (h), and Flake 3 (l), starting at the source electrode (0 μm) and ending at the drain (4 μm).

#### Flake 1

Under no tip bias, carrier density is highest at the electrode interface and lowest in the delaminated region inside the channel (Figure 5a). Raman analysis (E^1^_2g_ blueshift, Table 1) previously identified this region as one likely under compressive strain. Local strain changes the electronic structure, impacting the energy levels charge carriers can occupy.^56,57^ Under ±7 V tip bias (Figure 5b,c), carrier density increases within the delaminated region, suggesting it remains conductive but requires a larger driving force to populate energy states. A polarity-dependent response is observed: the MoS_2_/Au interface is depleted at +7 V but shows some carrier density at −7 V. A cross-sectional profile (Figure 5d) shows a spatial variation in polarity response, with depletion near the source at +7 V but enhanced signal after crossing the delaminated region. The opposite trend appears at −7 V.

#### Flake 2

The charge carriers within the defect of flake 2 follow a different trend (Figure 5e,f,g). The overall charge carrier density of the defect is highest under no DC tip bias and becomes depleted to different extents at different locations, depending on the polarity of the bias (Figure 5h). One explanation of the opposing trends from defects in flakes 1 and 2 relates to the different types of strain present. Flake 1 has an abrupt edge between the boundary of the basal plane and the topographically taller delamination defect that may pose as a barrier for charge-carrier transport into or out of the area (a location where ballistic scattering must be overcome). Material thinning is in-plane with the basal plane, which may allow for better lateral charge transport under no DC tip bias and thus a higher charge carrier density. The variation in signal between the basal plane and folded region may result from nonuniformity in electronic coupling between layers introduced at folding edges that is not seen in van der Waals stacking.^58^

#### Flake 3

Flake 3 has an intrinsic charge carrier density within the channel under no DC tip bias but is heavily depleted under DC tip bias, regardless of polarity (Figure 5i,j,l). However, the signal remains at the electrode interface.

The especially high signal at the MoS_2_-electrode interface of Flake 1, compared to Flakes 2 and 3, implies that the contact of Flake 1 with the electrode is different. The dC/dV amplitude signal was not sensitive to many of the topographical features, such as wrinkles, or to certain regions of high CPD, like the small blisters at the perimeter of Flake 1. It is known that the electric field is stronger at edges than on planes,^59^ which may explain why there is always a high charge carrier density where MoS_2_ overlaps with an edge of an electrode, even if it is depleted at other overlapping regions. It is interesting to note that the effect is stronger at the source compared to the drain, given that defects tended to grow near the source in a previous electronic stress study using monolayer MoS_2_.^9^

The polarity-dependent sMIM results for Flakes 1, 2, and 3 align well with their respective I–V behaviors. While the absolute tip voltage used in sMIM (±7 V) cannot be directly compared to the source–drain bias in Figure 2 because of the differing Schottky barriers at the tip–semiconductor and Au–semiconductor interfaces, the general trends in polarity-dependent responses are consistent. The I–V response for Flake 1 can be qualitatively summarized as having a strong response at negative voltages and a weak response at positive voltages (Figure 2a), and the sMIM results can be summarized as having charge carrier density across the MoS_2_ (within the channel and electrode interface) at negative voltages, and only within the channel at positive voltages. Reduced charge carrier density at the MoS_2_/electrode interface may explain the reduced I–V response at positive voltages. Flake 2 has carrier density across the MoS_2_ at both polarities, with some spatial variation due to material thickness changes. The overall intensity is higher at 7 V than −7 V, consistent with the magnitude of current at positive and negative voltages in the I–V response (Figure 2h). Lastly, Flake 3, which shows no I–V response, does not have high charge carrier density within the channel but does at the electrode interface independent of polarity, which may be key to understanding the loss of I–V behavior. However, we must exercise caution in generalizing the results here. Under tip bias, the tip-semiconductor junction can inject charge into the material^60^ rather than reporting on intrinsic charge carrier density, and the RF probes change in capacitance regardless of whether the flake completes a circuit (can pass current under bias between tip and electrodes). In Flake 3, the signal at the electrode interface rather than the channel implies the material is capacitively charging—not dissipating charge into the electrode. A disruption between the material and electrode could explain the loss in current response in the I–V sweep. We conclude it is necessary to have sufficient conductivity of the material both within the channel and at the electrode interface to maintain an I–V response after DC electronic stress.

These results underscore the importance of both in-channel and electrode-interface conductivity for device operation after electronic stress. sMIM, particularly with both AC and DC tip biases, could serve as a low-throughput screening tool for device viability, especially when combined with operando measurements. Future studies should explore whether microwave signals can assess the quality of electrode contact or predict failure by revealing early changes in local conductivity.

## Discussion

Detailed multimodal microscopy of three test structures provides insight into the morphological changes of MoS_2_ under DC electronic stress. These observations highlight potential problems in 2D material devices; however, they should not be interpreted as universally representative. Such overgeneralization from limited sample sizes remains a common limitation in the TMD literature over the past decade. For instance, it would be tempting (but misleading) to rationalize that delamination, thinning, or unchanged morphology are directly predictive of symmetric, asymmetric, or open-circuit I–V behavior. These three flakes were selected for multimodal characterization precisely because of their I–V responses, which biases interpretation. Indeed, only limited additional imaging is required to find counterexamples. As shown in Figure S11, two test structures both exhibit asymmetric I–V behavior after DC stress: one displays delamination, while the other shows no detectable morphological change at the basal plane or folded area, in contrast to the thinning observed in Flake 2.

To avoid adding narrowly observational results, here we focus on how both morphology and device-level geometry shape I–V performance and discuss how environmental factors complicate data interpretation. Additionally, sMIM reveals how stress-induced morphological features locally modulate charge carrier density. The results underscore the wide behavioral variability that arises even among nominally identical MoS_2_ test structures fabricated under the same conditions. Morphological change alone is not a reliable indicator of device degradation or I–V response. Still, SKPM can screen for morphological changes that impact electronic properties (as verified with sMIM), while mechanical measurements distinguish thinning from delamination. Chemical change, induced strain, or partial amorphization may increase resistance, but ultimately, the factors that dictate whether DC stress produces delamination, thinning, or no detectable change remain unclear.

### Possible Origins of Heterogeneous Morphological Changes

Expanded delamination observed in Flake 1 after electronic stress may be related to the limited overlap area between the flake and the electrode. MoS_2_ is known to delaminate from substrates under various forms of physical stress and strain.^26,61,62^ In this case, current was forced through a small contact area at the drain (0.02 ± 0.01 μm^2^), compared to a much larger area at the source (2.41 ± 0.01 μm^2^), likely creating a high local electric field sufficient to induce delamination. In transistor devices, such delamination can often be mitigated by applying dielectrics, or it may be inherently suppressed in vertical architectures/fully packaged devices where electrodes are deposited on top of the material. However, in devices that require the TMD surface to be exposed—such as gas sensors—this risk remains. For such applications, ensuring adequate high-quality overlap/contact between the TMD and electrode is critical to maintain device stability.

Material thinning surrounded by small beady features, as observed in Flake 2, has previously been reported in multilayer MoS_2_ subjected to electronic stress^12^ and ambient degradation.^63,64^ In the latter case, degradation has been attributed to the presence of O_2_ and H_2_O. However, the exact mechanisms underlying ambient degradation in TMDs remain under debate. Some studies suggest that O_2_ and H_2_O bind to chalcogen vacancies on the basal plane, promoting localized oxidation,^65,66^ while others propose that H_2_O intercalates from the flake edges, leading to material dissolution.^19,67^ In both scenarios, residual beadlike features (typically identified as transition metal oxides) are observed. Another explanation for this morphological change is a phase transition and/or dendritic growth due to the electromigration of metal between the electrodes. Regardless of the specific pathway, these mechanisms must be considered here, given that the observed morphological thinning due to electronic stress under ambient conditions occurred both here and in the study by Pezoldt and coworkers.^12^ There is no evidence of a new crystalline phase in the Raman spectra (Figure 3d), although an amorphous phase without Raman-active modes cannot be ruled out. The thinned area cannot be metallic because Flake 2 showed no signs of open-circuit behavior after electronic stress (Figure 2h), which also rules out dendritic growth of the electrode across the channel.

This begs the question: why would flakes 2 and 3 behave differently despite apparently sufficient contact with electrodes (similar I–V behavior before electronic stress)? One explanation is a variation in defect density, like S vacancies, at the surface. Chandross and coworkers computationally demonstrate that H_2_O strongly binds to S vacancies on interlamellar surfaces, with one H_2_O per vacancy.^66^ Thus, areas of high S vacancy concentration on the surface would be more prone to material thinning by dissolution. To test the probability of this hypothesis, spatially resolved X-ray photoemission spectroscopy (μXPS) was done using X-ray photoemission electron microscopy (XPEEM) at the ESM (21-ID) beamline of NSLS-II to determine if large regions of S vacancies are heterogeneously distributed within individual flakes. Figure 6a shows a μXPS image of the S 2p for four individual MoS_2_ flakes as transferred. Here, higher pixel intensity indicates a stronger S 2p signal. Three flakes have some regions of lower pixel intensity (indicated with yellow arrows) within them, which implies either less S or some kind of contamination on the surface of the flake attenuating the signal. A μXPS image of the C 1s peak was taken as a control because C is the most likely surface contaminant (Figure 6b). However, the μXPS image of C 1s does not show any features that correlate with the low-intensity S 2p regions (Figure 6)—the low-intensity S 2p signal may indicate S vacancies in three of the four imaged flakes. A heterogenous distribution of S vacancies could explain why Flake 2 exhibits thinning while Flake 3 does not.

**Figure 6:**
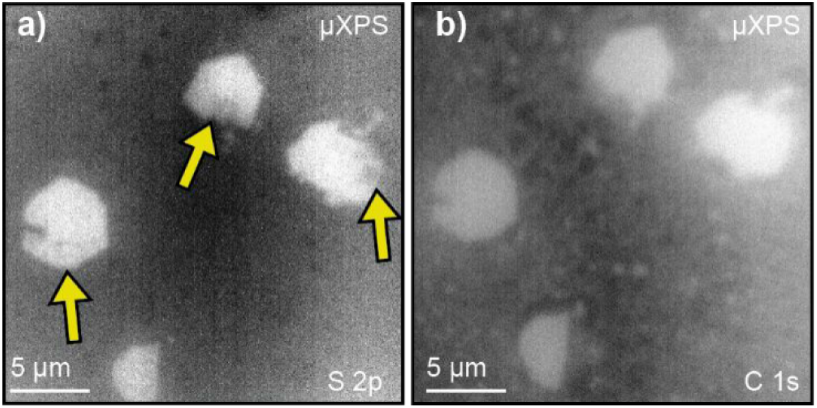
μXPS from XPEEM on PMMA-transferred MoS_2_flakes. a) μXPS of S 2p and (b) C 1s. Yellow arrows guide the eye to locations of lower contrast on the S 2p image.

Neither low-energy electron reflectivity (LEER) nor SKPM showed any correlation of work function or CPD, respectively, within the low-intensity S 2p regions (Figure S12), which means screening for these defects would be challenging if they prove to be a culprit of poor device behavior. Different microscopies are needed to screen for high concentrations of S vacancies on the surface.

μXPS is more sensitive than Raman. The strong C 1s signal observed in μXPS provides evidence of environmental contamination, but no vibrational modes associated with carbon species were observed in Raman (Figure S5). The C 1s peak is centered near 284 eV, characteristic of C–C bonding (Figure S13), and is consistently detected across the sample. Localized regions of elevated signal between MoS_2_ flakes (not present in the S 2p data) suggest residual surface contamination, most likely from PMMA used during transfer. The same acetone and IPA cleaning procedure was used on all samples prior to any measurement but is insufficient to remove residue. PMMA residue is a known issue that causes variability in performance^13^ and likely plays a role here. In addition, we postulate that surface adsorbents induce redox activity both before and after DC stress, which complicates fair comparison of devices (and therefore fundamental understanding of mechanisms that lead to morphological change).

Atomistic modeling could provide additional mechanistic clarity regarding the stress-induced phenomena observed here. Density functional theory studies have shown that uniaxial or biaxial strain modifies the MoS_2_ band structure and work function,^56,57^ consistent with the CPD shifts observed in delaminated regions. A representative CPD shift of ∼20 mV corresponds to an effective trapped charge density on the order of 10^9^–10^10^ cm^–2^ (assuming 300 nm SiO_2_ see SI for calculations (equations S1–S4), which lies within the range of interface and adsorbate trap densities commonly reported for MoS_2_ devices (10^10^–10^12^ cm^–2^).^17,68,69^ Similarly, theoretical work demonstrates that sulfur vacancies strongly influence adsorption energetics of H_2_O and O_2_,^66^ suggesting that vacancy heterogeneity may predispose certain regions to thinning or degradation under ambient biasing conditions. Modeling of metal–MoS_2_ interfaces further indicates substantial charge transfer and band bending at contacts,^48^ which aligns with our observation that poststress I–V behavior correlates more strongly with electrode geometry than with visible morphology. While such modeling would refine quantitative interpretation (e.g., predicted band shifts, vacancy formation energies, or adsorption barriers), it would not be expected to fundamentally alter the key experimental conclusion of this work: that stress-driven structural evolution and device-level variability are heterogeneous and strongly contact-dependent. Future efforts combining operando measurements with multiscale modeling may help disentangle strain, defect chemistry, and contact effects more precisely.

## Conclusions

Low-complexity test structures were fabricated by transferring multilayer MoS_2_ flakes onto prefabricated gold electrodes. These devices exhibited a wide range of current–voltage (I–V) responses—near-Ohmic, rectifying, and open-circuit response. Devices subjected to electronic stress under a 20 V DC bias yielded three distinct outcomes: (1) a uniform increase in maximum current (i_max_), (2) an asymmetric, polarity-dependent response with both increased and decreased i_max_, and (3) a complete loss of I–V response.

To investigate the origin of these behaviors, we performed multimodal characterization, including SEM, AFM, SKPM, Raman spectroscopy, and sMIM, on an apparently representative MoS_2_ flake from each category. We find that electronic stress drives diverse structural and electronic changes, including delamination and material thinning, which can be distinguished by variations in mechanical stiffness and Raman signal. The correlation between morphology and I–V response is nontrivial despite these clear morphological signatures: sMIM reveals that stress-induced features reduce local carrier density, yet one device with complete electrical failure showed no discernible morphological change within the resolution limits of our measurement tools. We speculate that changes in strain from delamination play a role in the increased current response for Flake 1, and a combination of material thinning (reduced layer number) and amorphization contributes to the asymmetric current in Flake 2. These results cannot be generalized to all flakes displaying those outcomes; additional imaging showed flakes with asymmetric I–V response can have signatures of delamination or no morphological change. No universal correlations emerged between morphology and electrical response. Device geometry (electrode overlap, channel area, and contact length) proved to be a stronger predictor of poststress behavior than morphology.

Environmental contamination is a confounding variable in this study: I–V behavior change might arise not just from purely electrical or structural changes internal to MoS_2_, but also from interactions with surface-adsorbed contaminants. This underscores the importance of controlling conditions during device construction and testing. Using surface passivation or encapsulation is not always practical when the TMD must be directly probed, and monitoring chemical composition (e.g., via XPS or other surface-sensitive tools) alongside morphological and electrical measurements drastically increases experimental intricacy. These results underscore the complexity of stress-driven change in MoS_2_ and highlight the need for correlative, high-resolution imaging approaches to link charge transport behavior with structural evolution and to assess the quality of electrode contact. Small-sample-size experimental schemes can provide useful information but should not be relied upon to reveal a wholly representative understanding. We hope this work motivates large-scale reliability studies and catalyzes new efforts to uncover the fundamental mechanisms by which TMDs change under operational conditions and serves as an additional warning against the potential convolution of results caused by environmental contamination of samples. Processing, environment, and test protocol are not background noise; they are core variables. To make reliable, generalizable claims about stress-driven changes in MoS_2_, studies must couple multimodal, surface-sensitive characterization with deliberate environmental control, explicit contact metrics, and statistically meaningful device populations.

## Methods

### Material Syntheses by CVD

MoS_2_ multilayer flakes were synthesized in an SH Scientific single-zone tube furnace (model SH-FU-50LTH-WG) with a 600 mm heating zone and a 50 mm diameter. The gas flow was controlled by Alicat Mass Flow Controllers, and the furnace was purged using an SH-VBD10 vacuum pump. The reaction was carried out in a quartz tube connected to a 99.99% pure argon cylinder.

Two ceramic boats (MSE Supplies) were loaded with 1000 mg of sulfur (99.9995% pure by metals basis, Thermo Scientific) and 50 mg of MoO_3_ (99.9995% pure by metals basis, Alfa Caesar) powder. Silicon wafers (2 cm × 1 cm) with 300 nm SiO_2_ (MSE Supplies) were cleaned by sonication in acetone for 5 min, followed by an isopropyl alcohol rinse. The wafers were placed between 3 and 5 cm downstream from the MoO_3_ boat in the quartz tube furnace, with the sulfur boat positioned approximately 20 cm upstream relative to the wafers. The system was purged three times with argon before heating to 850 °C at 13 °C/min, maintaining the temperature for 10 min, and then cooling to room temperature by natural convection. The argon flow rate was 100 cm^3^/min.

### Device Fabrication and Material Transfer

SiO_*x*_/Si wafers were cut into ∼3 mm^2^ chips fabricated with Ti/Au (5 nm/95 nm) electrode pairs with a 4 μm gap using a metal lift-off process. Then, MoS_2_ on SiO_*x*_/Si wafers was sectioned into ∼3 mm^2^ squares using a diamond scribe. Poly­(methyl methacrylate) suspension in anisole (PMMA, 950 A3, Kayaku) was spun onto the MoS_2_ surface at 1000 rpm for 60 s and then 2000 rpm for 30 s, dried on a hot plate at 80 °C for 30 min, and placed under vacuum overnight.

The PMMA-coated wafer was transferred to the target substrate with electrodes using a wet liftoff.^23,24^ Briefly, the edges of the PMMA were removed using a scalpel and then submerged in 30 wt % KOH at room temperature until the PMMA lifted off (>30 min). The PMMA/MoS_2_ was then transferred to clean DI water for 10 min (3 times) before using the target substrate to scoop and complete the transfer. The PMMA/MoS_2_/target substrate was dried on a hot plate at 80 °C for 30 min and placed under vacuum overnight. PMMA was removed by submerging the sample in acetone for ∼30 min. This transfer process was used for both electronic test structures and TEM grids and yields randomly dispersed MoS_2_ flakes across the substrate. Only electronic test structures comprised of Ti/Au electrode pairs bridged by a single MoS_2_ flake are considered in this study.

### Material Characterization

MoS_2_ Raman spectra were taken with a Renishaw inVia confocal Raman microscope using a 532 nm laser focused through a 100X objective. An IMA Hyperspectral Microscope from Photon etc. was used to take photoluminescence spectra under 532 nm laser excitation. A Zeiss Gemini 300 Variable Pressure SEM or Zeiss LEO 1525 SEM was used for electron imaging (2 −5 keV). Transmission electron diffraction patterns were recorded using a digital camera (Thorlabs CS135MU) in the SEM (20 keV), as described elsewhere.^70^ Micro-Raman mapping was conducted on a HORIBA LabRam HR Evolution confocal microscope using 532 nm excitation and a 1,800 lines per mm grating. Laser power was limited to 300 μW to prevent laser-induced damage to the MoS_2_ flakes. A set of three volume Bragg gratings was placed in the excitation path to resolve the low-frequency region of the Raman spectrum. The Raman spectra presented in the SI were taken in resonance at 633 nm.

### Scanning Probe

A Bruker Icon Atomic Force Microscope was used to perform nanomechanical, topographic, and surface potential measurements. PeakForce imaging mode was used for the simultaneous acquisition of topography and mechanical modulus. Scanning Kelvin Probe Microscopy (SKPM) was used for simultaneous topographic and surface potential measurements. SKPM measurements were performed using a nominal 2.8 N/m cantilever (NanoWorld, EFM-50) with Pt/Ir coating for conductivity (resonance frequency ≈ 70 kHz). SKPM measurements were performed in dual-pass, amplitude-modulation mode with a typical lift height of 15 nm relative to the tapping mode first pass. A first-order plane fit was used to postprocess height maps. The SKPM shown is unprocessed data, arithmetically normalized to the average signal from Au. Mechanical measurements were taken in the PeakForce scanning mode using a nominal 40 N/m cantilever (Bruker, RTESPA-300–30). Topography was normalized with a plane fit, and Force–Distance curves were fit with a line.

Scanning microwave impedance microscopy (sMIM) measurements were performed with an Asylum MFP-3D Atomic Force Microscope equipped with a PrimeNano ScanWave Pro add-on and SMIM-150 μm cantilever. Scans were done in contact mode with driving AC voltages ranging from 100 mV up to 3 V. The tip bias voltages ranged from 0 V to ±7 V, keeping the total AC plus DC voltage within the maximum of ±10 V. The RF frequency was kept constant at 2.97 GHz. Following the channel identification procedure based on a commercial Al chip with deposited micrometer-scale capacitances suggested by the manufacturer allows the separation of the low-frequency modulated RF signal to be decomposed into resistive and capacitive components in the I/Q mixer. Scanning over the “ideal” capacitance on the chip allows to adjust the phase of the reference RF I/Q mixer signal in such a way that the signal corresponding to the real part of the complex response in one of the displayed imaging channels, the resistive channel, is set to zero, and all the response is displayed in the capacitive channel. This approach allows one to simplify the interpretation of the observed images and better understand the underlying physics. All sMIM presented image data were processed by leveling data by a first-order mean plane subtraction and by removing the first-order polynomial background.

### Electronic Analysis

Current was measured using a two-point measurement for both constant DC voltage and voltage sweeps, using a Signatone S-1160 probe station and a Keithley 2420 source meter by landing one probe on each of the Au pads that comprised the MoS_2_ test structure under ambient conditions. Much of the electronic testing was done on the Bruker Ion AFM by landing probes on the sample being analyzed by AFM (see Figure S14). Linear or cyclic sweeps were done between −20 and 20 V. Here, we define near-Ohmic as an I–V trace that can be fit to a line with an R^2^ > 0.95. The typical length of DC stress measurements were ∼1 h. A 20 V DC bias was chosen because it is on the lower range of potentials shown to cause defect growth (20 V for 96 h);^9^ less time was required here. Other studies report lower potentials for defect growth but also apply gate voltages.^11,12^ Current–voltage (I–V) curves taken after DC stress were measured every 3 min until the data was consistent for 15 min. The typical delay between the end of the DC voltage pulse and the I–V measurements displayed in this manuscript are ∼45 min. This equilibration time is needed because the MoS_2_ test structures capacitively charge.^71^ Mild hysteresis was observed between forward and backward sweeps, consistent with charge trapping and adsorbate effects widely reported for MoS_2_ devices on SiO_2_ substrates under ambient conditions.^16,17^ Forward (V_f_) and reverse (V_r_) onset voltages were determined from the first derivative (dI/dV) of the I–V curve, where the inflection point corresponding to the most rapid increase in conductance was identified. This approach was selected to provide a consistent comparative metric across nonideal metal–semiconductor–metal devices.

### Beamline Experiments

Beamline experiments were carried out at the Electron Spectro-Microscopy (ESM, 21-ID) beamline at NSLS-II. Flakes of MoS_2_ were transferred to Si wafers with 1–3 nm native oxide, as described in Device Fabrication and Material Transfer. Thin native oxides are necessary to ensure sufficient conductivity of the surface and to mitigate potential surface charging detrimental to LEEM/XPEEM measurements. The sample was annealed at 300 °C in ultrahigh vacuum for 12 h before analysis. Selected area low-energy electron diffraction (μLEED) was done using a 1.5 μm sampling area while sweeping the voltage from 30–80 eV in 1 eV steps. Low-energy electron microscopy was done using either a 20 or 40 μm field-of-view from −4 to 6 eV in 0.1 eV steps. X-ray photoemission electron microscopy (XPEEM) was used to achieve spatially resolved X-ray photoelectron spectroscopy (μXPS) images of the S 2p by acquiring data at a binding energy of 162 eV (83 eV kinetic energy) using photon energy of hν = 250 eV. The C 1s μXPS image was taken at a binding energy of 284 eV (61 eV kinetic energy) and photon energy hν = 350 eV, with an analyzer slit of 2 eV, centered over the corresponding XPS peak.

## Supplementary Material



## Notes

Additional experimental details; materials and methods, including photographs of the experimental setup; correlations; trapped charge calculations; and current–voltage examples (PDF)
