# Supplementary material for: Characterization of Electronic Stress-Induced Changes in Multilayer MoS2
Source: ACS Appl Electron Mater. 2026 Apr 6;8(8):3516–29. doi: 10.1021/acsaelm.6c00080 (PMC13130964; doi:10.1021/acsaelm.6c00080)
Supplement: Supplementary file 1 [file el6c00080_si_001.pdf]

Supporting Information:

Characterization of Electronic Stress-Induced Changes in Multi-Layer MoS_2

R. Colby Evans[a,], Riccardo Torsi[b], Pavel Kabos[c], Jason Holm[a], Jason P. Killgore[a], Paul Owiredu[d], Gurpreet Singh[d], Jerzy T. Sadowski[e], Angela R. Hight Walker[b], and Elisabeth Mansfield[a,*]

Certain commercial equipment, instruments, or materials are identified in this report to specify the experimental procedure adequately. Such identification is not intended to imply recommendation or endorsement by the National Institute of Standards and Technology, nor is it intended to imply that the materials or equipment identified are necessarily the best available for the purpose.

[a] National Institute of Standards and Technology, Applied Chemicals and Materials Division, Boulder, CO, 80305, United States.

[b] National Institute of Standards and Technology, Quantum Measurement Division, Gaithersburg, MD, 20899, United States

[c] National Institute of Standards and Technology, Applied Physics Division, Boulder, CO, 80305, United States.

[d] Department of Mechanical and Nuclear Engineering, Kansas State University, Manhattan, Kansas 66506, United States.

[e] Center for Functional Nanomaterials, Brookhaven National Laboratory, Upton, New York 11973, United States.

**Corresponding author email: Elisabeth.Mansfield@nist.gov*

Synthesis Discussion....................2

Other Characterization....................5

Correlations....................7

More Asymmetric I-V Change Examples....................11

XPEEM, AFM, SKPM....................12

Experimental setup....................14

Bibliography....................14

Synthesis Discussion

Figure S1 shows a scheme for material synthesis and subsequent structural and spectroscopic characterization of the resulting multilayer MoS_2. Multilayer MoS_2 was synthesized by chemical vapor deposition (CVD) using MoO_3 and S as precursors in a quartz tube furnace (Figure S1a; see Methods for details).These growth conditions were optimized from literature[1–4] to facilitate uniform growth of > 5 µm MoS_2 flakes across the entire wafer surface. The ratio of Mo to S precursors as well as the Ar flow rate had pronounced effect on the flake size and substrate coverage—MoS_2 flakes were relatively small (ranging approx. 2 to 5 um) and fewer (low substrate coverage, approx. 10%) for Ar flow of 300 sccm while the higher Mo/S precursor mass ratio (e.g. >20 and up to 40) mostly lead to formation of thick particle-type morphology. Further, the growth times longer than 10 min did not seem to produce flakes with larger average lateral size and/or continuous films.[5] Scanning electron microscopy (SEM) imaging revealed a collection of flakes with heterogeneous geometries, ranging from regular hexagons to irregular polygons, all maintaining 3- or 6-fold symmetry (Figure S1b). Some as-grown flakes show signs of folding back on top of themselves. A representative SEM image of a single MoS_2 flake is shown in Figure S1c, accompanied by its hexagonal electron diffraction pattern in Figure S1d. The 3-fold crystalline symmetry of the material was confirmed by selected area low energy electron diffraction (µLEED) analysis (Figure S2).

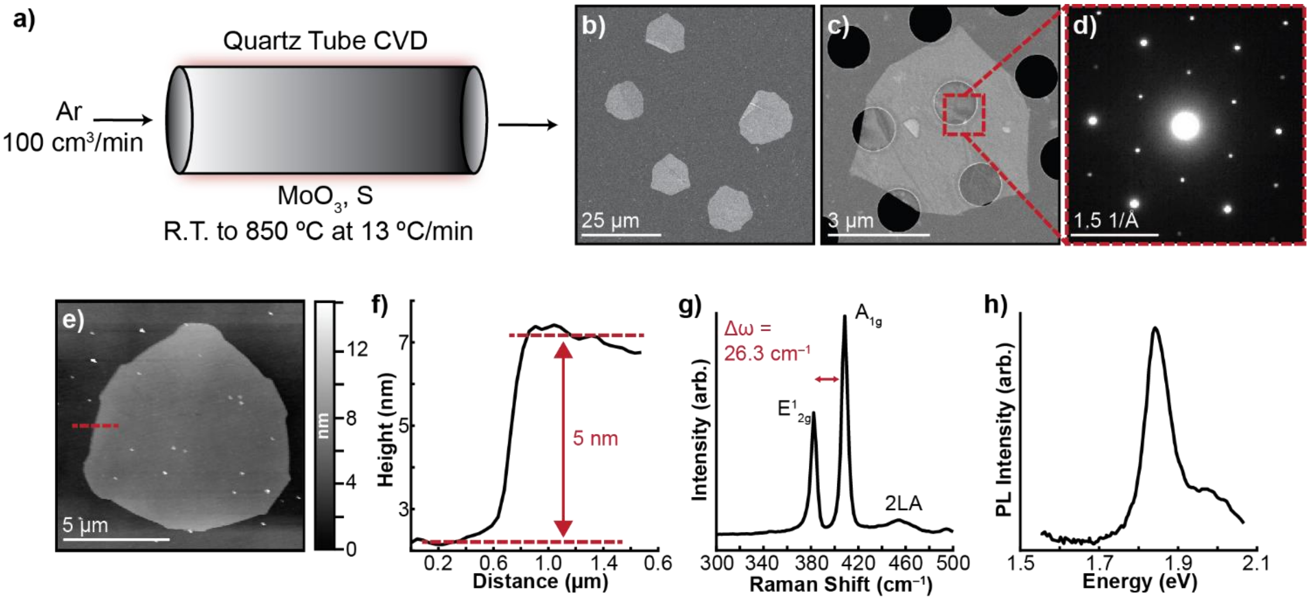

Figure S1 – Materials synthesis and characterization. a) scheme of the MoS_2 synthesis using chemical vapor deposition. b) SEM image of the resulting growth on SiO_x/Si. c) SEM image of a MoS_2 flake transferred to a Quantifoil grid and d) its respective diffraction pattern. e) AFM image of a flake on SiO_x/Si growth substrate and the f) resulting height map along the region of interest (red line in e). g) Raman spectrum and h) photoluminescence spectrum.

Topographical characterization using atomic force microscopy (AFM) is shown in Figure S1e, where a height map (Figure S1f) of a representative flake on the growth substrate confirms the multilayer structure of the synthesized material. Raman spectroscopy (Figure S1g) supports the identification of multilayer MoS_2, as indicated by the difference in wavenumber, Δω, between the E^1_{2g} and A_{1g} vibrational modes, which exceeds ~21 cm^{-1}; the second order longitudinal acoustic peak at ~ 450 cm^{-1} corresponds to a phonon mode.[6,7] Photoluminescence spectroscopy (Figure 1h) shows emission peaks at 1.85 eV and 1.95 eV, consistent with A and B excitons characteristic of hexagonal MoS_2.[8]

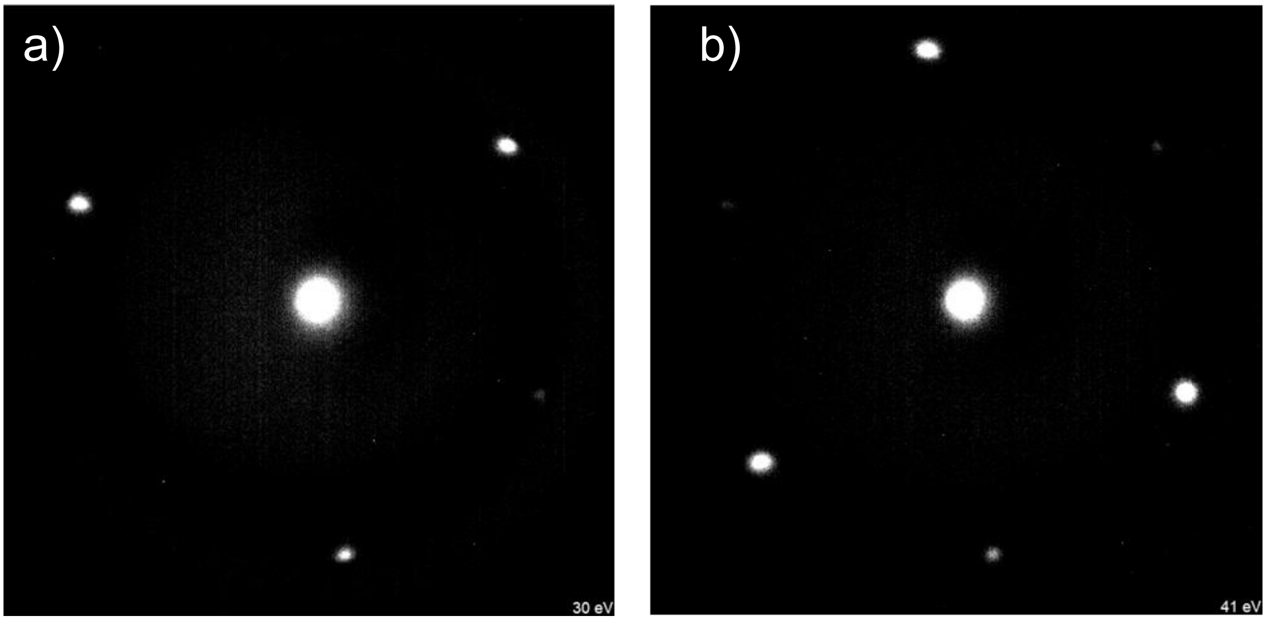

Figure S2 - The 3-fold crystalline symmetry of the material was confirmed by selected area low energy electron diffraction (µLEED) analysis.

Interparticle heterogeneity was evaluated by analyzing the work function of nine individual MoS_2 flakes using low energy electron microscopy (LEEM). The 9 flakes consisted of two areas located a few millimeters away to ensure different growth regions were evaluated. Area 1 had six flakes and Area 2 had three. Area 1 is shown in a mirror-mode LEEM image in Figure S3a. The MoS_2 flakes appear darker (lower pixel intensity) due to a difference in electron reflectivity between them and the substrate, at the specific energy. The low energy electron reflectivity (LEER) spectra obtained from the flakes can provide information on the local surface potential. The spectra can be simultaneously acquired by conducting dynamical measurements in LEEM, in which the incident electron energy is tuned, and the corresponding reflected electron intensity is recorded in the form of images of the surface (LEEM-IV).[9] The energy at the onset of a drop in reflectivity of the electrons can be used to estimate local differences in surface potential (work function). Figure S3b,top shows the extracted mean pixel intensity for the MoS_2 flake labeled "I". Taking the derivative (Figure S3b,bottom) yields the onset of the drop in electron reflectivity. Figure S3c shows a histogram for all nine flakes. The distribution for the onset of the drop in reflectivity is within 0.2 eV ± 1 eV which indicates very little difference. We note here that the absolute value of the energy shown in Figure S3c were extracted from mirror-LEEM images taken at Brookhaven National Lab – absolute energies are not consistent across microscopes due to tuning of electron optics or different in electron gun and sample potentials. Mirror-LEEM was chosen to provide a contrast between the flakes and the substrate to look for relative differences across the sample, not to quantitatively extract work functions.

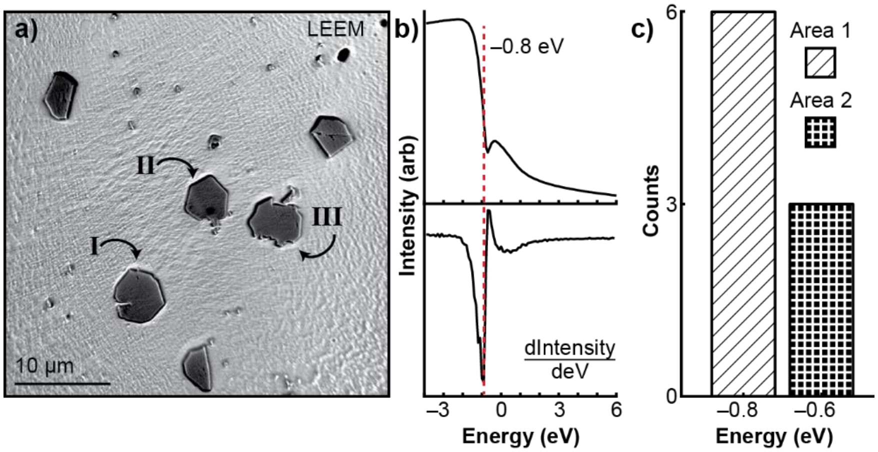

Figure S3 – LEEM and XPEEM data. A) mirror-mode LEEM image at of MoS_2 flakes (darker contrast). b,top) Intensity profile as a function of energy for flake "I" and its derivative (b,bottom). The red dashed line indicates the point of inflection that defines the surface potential (proportional to work function) for each flake. c) a histogram of surface potentials for 9 flakes over two areas.

Other Characterization

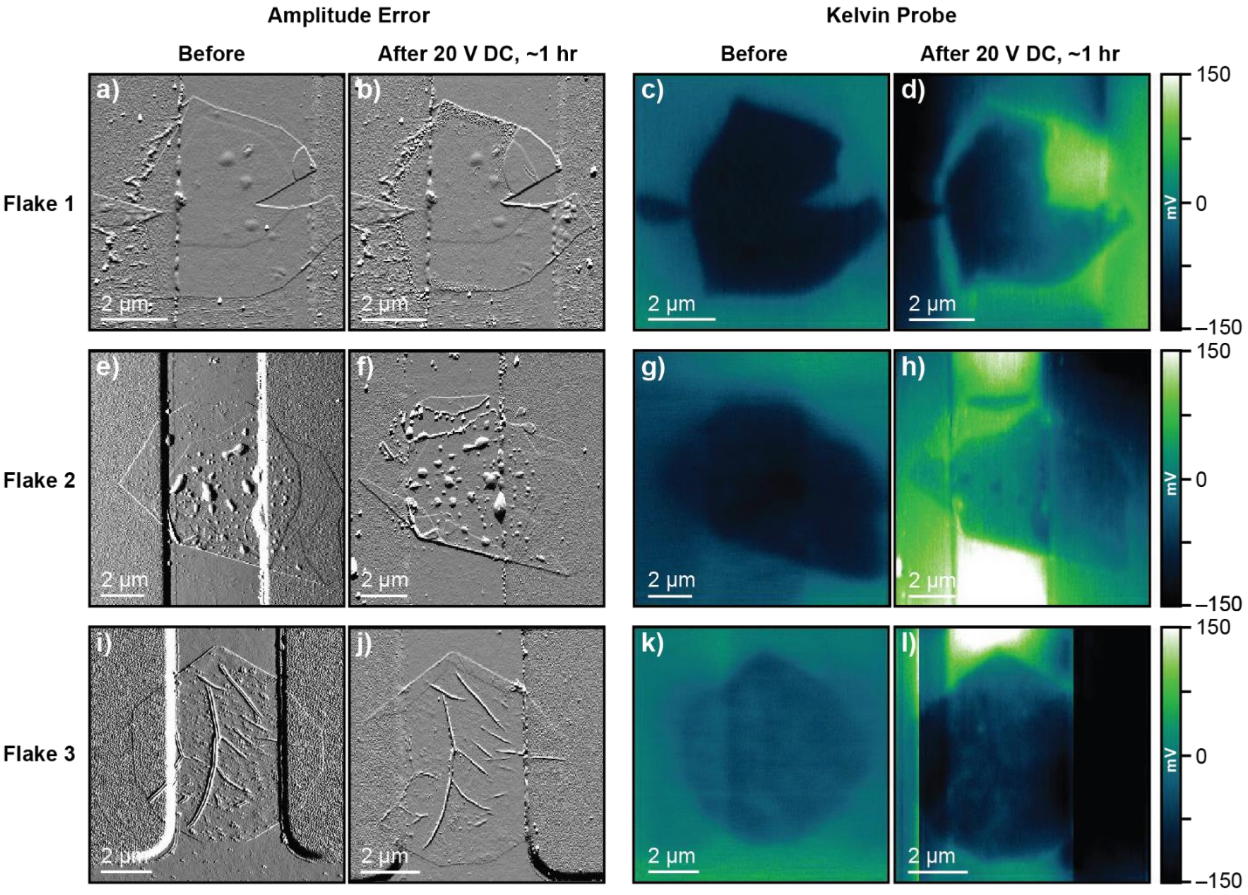

Figure S4 – Edge detection and impact of defects on contact potential difference (CPD). (a,e,i) Error signal from the cantilever amplitude signal and (c,g,k) CPD from Kelvin probe before DC electronic stress for Flakes 1, 2, and 3, respectively. (b,f,j) Error signal from the cantilever amplitude signal and (d,h,l) CPD from Kelvin probe after 20 V DC electronic stress for Flakes 1, 2, and 3, respectively. CPD was normalized to the average of the electrode signal to compare MoS_2 scanned with different cantilevers.

Amplitude error (derived from AFM scans) acts as an edge-detection signal to more clearly highlight topographical changes within the MoS_2 on electrodes and in the channel (Figure S4a,b). Before stress, Flake 1 exhibits several blisters and a semi-circular edge connecting an interior flake to the drain electrode above a wedge-like tear. After stress, three new features emerge: (1) expansion of the semi-circular structure, (2) new blister-like features over the drain electrode, and (3) bump-like features at the channel edges. All appear as high-CPD regions absent prior to stress (Figure S4c,d). Additionally, the basal plane CPD increases by 21.3 mV ± 15.3 mV, consistent with either p-type defect doping or increased strain.[10–12] These localized CPD changes reflect altered electronic structure or trapped charge. Flake 2 contains many blisters in a folded region of the flake. After stress, a thin feature spanning the channel grows from ~100 nm at the drain to ~1 μm at the source, bounded by the step edge from the intra-flake fold (Figure S4e,f). New blisters form at electrode interfaces, and existing blisters split into multiple circular domains. CPD increases across the basal plane and the newly formed features show distinct CPD contrast (Figure S4g,h). The area corresponding to material thinning shows increased CPD consistent with increased strain.[10] Three low-CPD regions appear post-stress implying to formation of more strongly n-doped regions.[11] For Flake 3 some small surface features disappear, and a central wrinkle flattens.

While no prominent topographic changes are detected in amplitude error, CPD becomes more heterogeneous and drops by 27.6 mV ± 23.0 mV. The elevated and variable CPD in the unstressed state may indicate a higher intrinsic disorder (heterogenous distribution of mid-gap states) compared to Flakes 1 and 2.[12]

Stress-induced carrier trapping at the MoS_2 surface or MoS_2/SiO_2 interface is a plausible contributor to the observed CPD shifts.

To provide a quantitative estimate, we approximate the CPD ($\Delta V = 20\ mV$) change as arising from an effective trapped surface charge density using equations S1-4:

$$\Delta V = \frac{\sigma}{C_{ox}} \qquad S(1)$$

$$C_{ox} = \frac{\varepsilon_0 \varepsilon_r}{t} \qquad S(2)$$

For t = 300 nm SiO_2, assuming the charge is trapped between the MoS_2 and dielectric, ($\varepsilon_r \approx 3.9$, “Silicon Processing for the VLSI Era", Stanley Wolf and Richard N. Tauber, Lattice Press, 1986), $C_{ox} \approx 1.1 \times 10^{-8}$ F/cm^2.

Convert to carrier density:

$$n = \frac{\sigma}{q} \qquad S(3)$$

$$n \approx \frac{2.3 \times 10^{-10}}{1.6 \times 10^{-19}} \approx 1.4 \times 10^{9}\ \text{cm}^{-2} \qquad S(4)$$

A representative CPD shift of ~20 mV therefore corresponds to an effective trapped charge density of approximately 10^9–10^{10} cm^{-2}. This value lies within the range reported for interface trap densities in MoS_2 devices under ambient conditions[13–16] supporting the plausibility of stress-induced charge trapping.

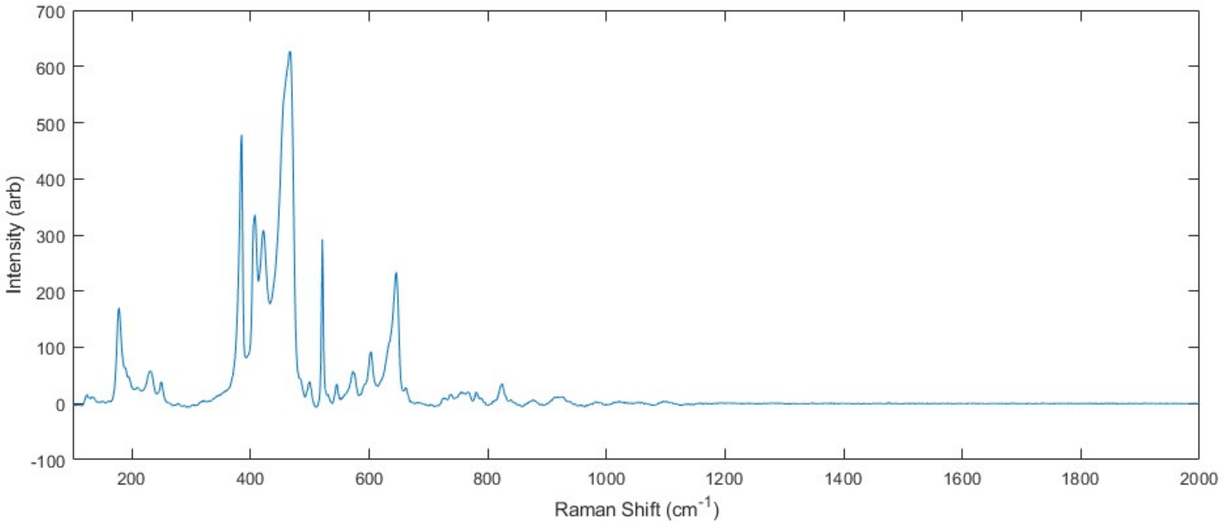

Figure S5 – Resonance Raman spectra of an MoS_2 flake between Au pads taken at 633 nm.

There are no clear Raman peaks for oxidation products or C-based contamination (Figure S5).[17–19] This could be in part to the large PL background that needed to be subtracted, however all peaks correspond to known MoS_2 modes.[20–22]

Correlations

As Prepared	V_f	V_r	$Log(i_f)$	$Log(i_r)$
V_f	-	-	-	-
V_r	0.47 ± 0.1	-	-	-
$Log(i_f)$	-0.03 ± 0.1	0.05 ± 0.1	-	-
$Log(i_r)$	-0.10 ± 0.1	0.03 ± 0.1	0.76 ± 0.04	-

Table S1 – Pearson's correlation coefficients between I-V parameters of as prepared device. Weak correlations are colored yellow (> 0.30 ± 0.1 < 0.6 ± 0.1) and strong correlations are colored green (> 0.60 ± 0.1).

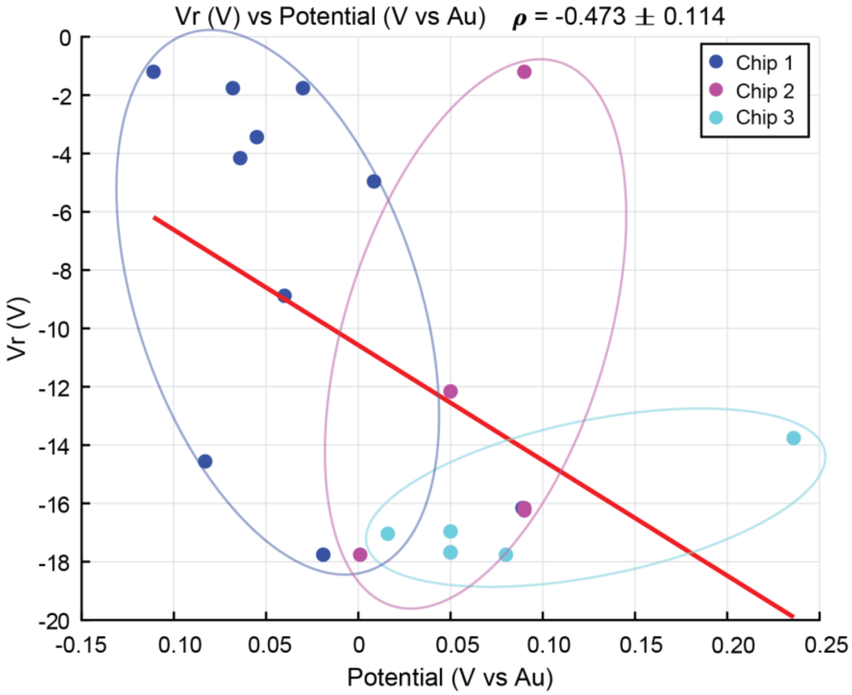

Figure S6 – Correlation of CPD corrected to Au electrodes vs reverse bias threshold (V_r). The linear fit and circles are present to guide the eye

Figure S6 shows a representative correlation where there is chip-dependent clustering. Circles around qualitative clusters are present to guide the eye. This chip dependent clustering is present throughout correlation plots before DC stress. The same weak anticorrelation is not present within individual chips, but because of the particular spread of data there is a false correlation that appears when they are graphed together.

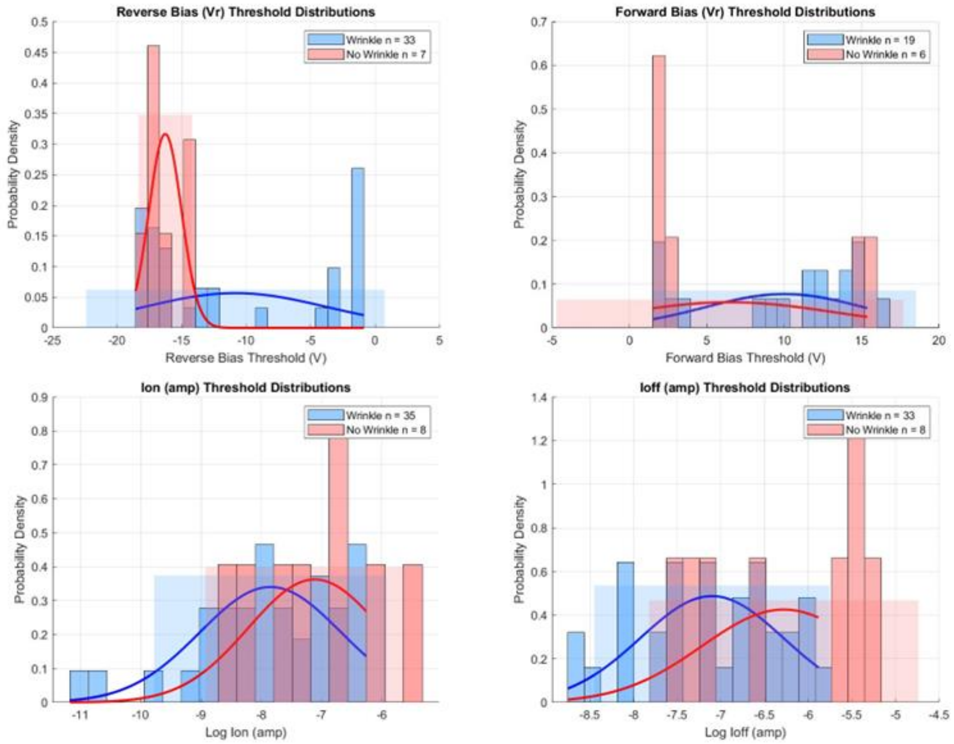

Figure S7 – Overlay of histograms for I-V metrics and presence (blue) or absence (red) of wrinkles. Gaussian fits are present to guide the eye; distributions are not normal. Translucent areas represent 90% confidence interval for the data.

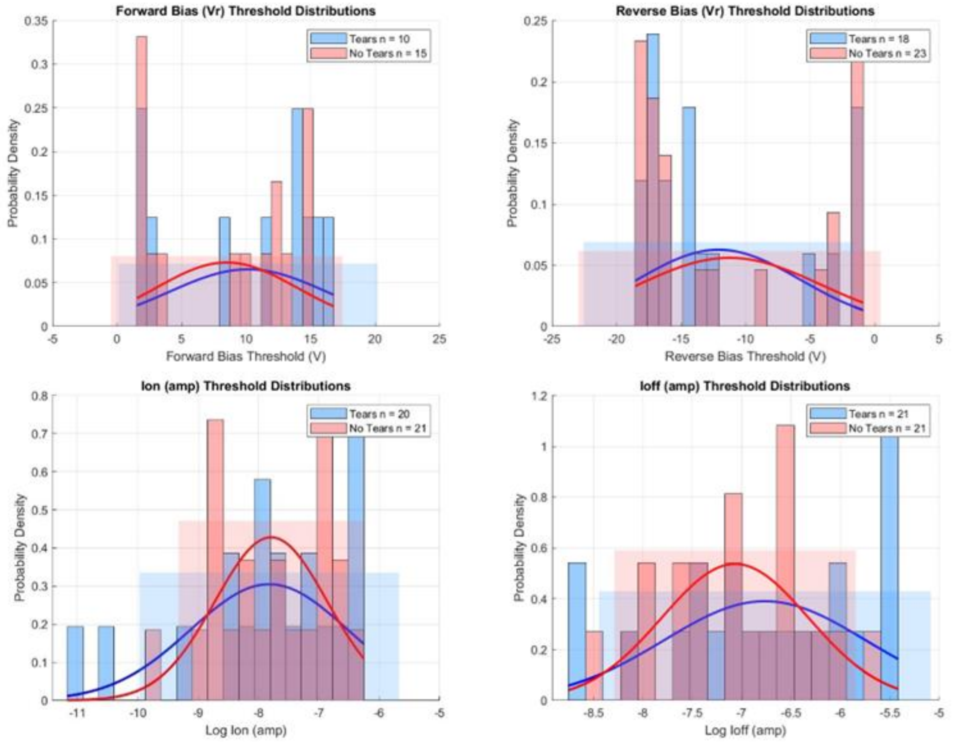

Figure S8 – Overlay of histograms for I-V metrics and presence (blue) or absence (red) of tears. Gaussian fits are present to guide the eye; distributions are not normal. Translucent areas represent 90% confidence interval for the data.

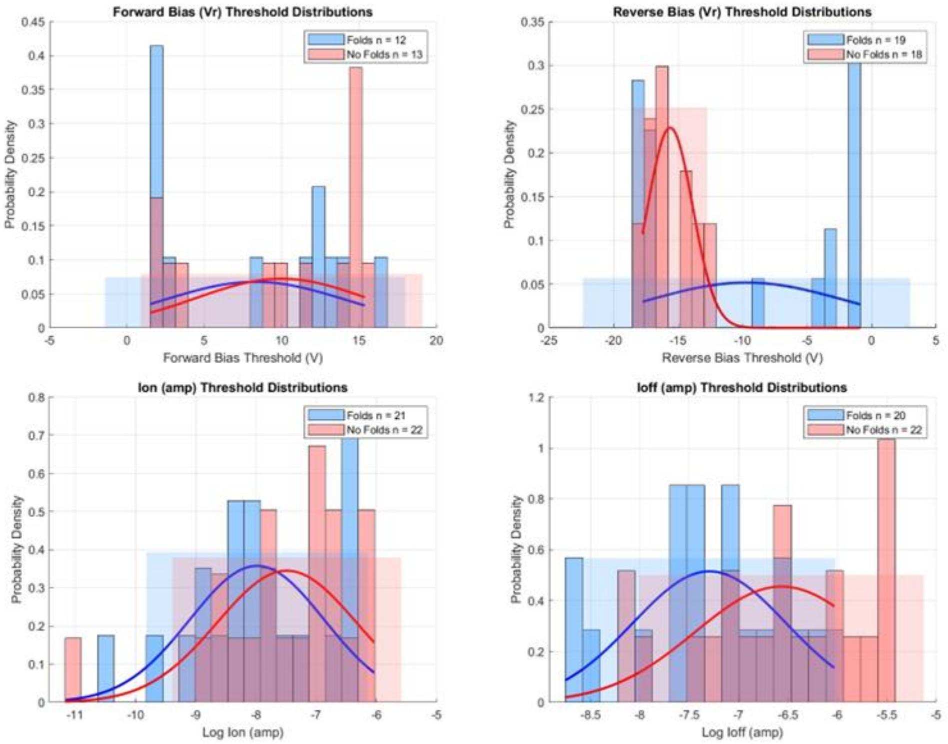

Figure S9 – Overlay of histograms for I-V metrics and presence (blue) or absence (red) of folds. Gaussian fits are present to guide the eye; distributions are not normal. Translucent areas represent 90% confidence interval for the data.

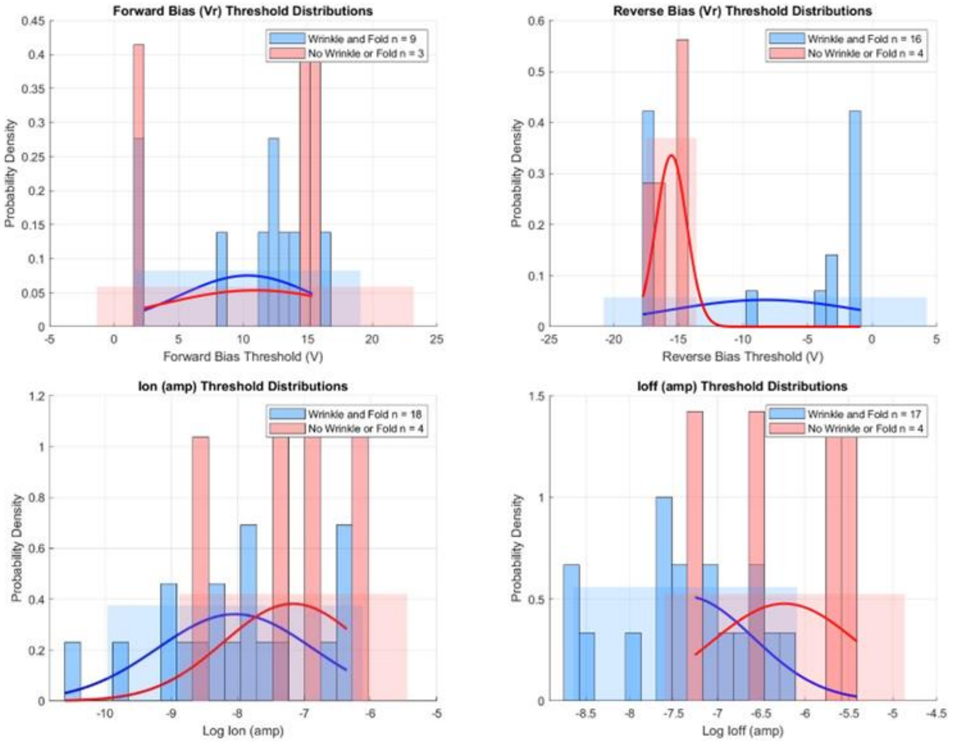

Figure S10 – Overlay of histograms for I-V metrics and presence (blue) or absence (red) of wrinkles and folds. Gaussian fits are present to guide the eye; distributions are not normal. Translucent areas represent 90% confidence interval for the data.

Figures S7-10 show histograms for I-V metrics and one of several morphological changes (wrinkles/no wrinkles, tears/no tears, folds/no folds, and wrinkles and folds/ no wrinkles or folds). Distributions are not normal, but fit with a Gaussian to guide the eye. Translucent areas represent 90% confidence interval. Probability density indicates the likelihood of an instance within a population, rather than raw counts at a value showing distribution. Overlapping probability densities indicate that the populations do not have significantly different behavior.

More Asymmetric I-V Change Examples

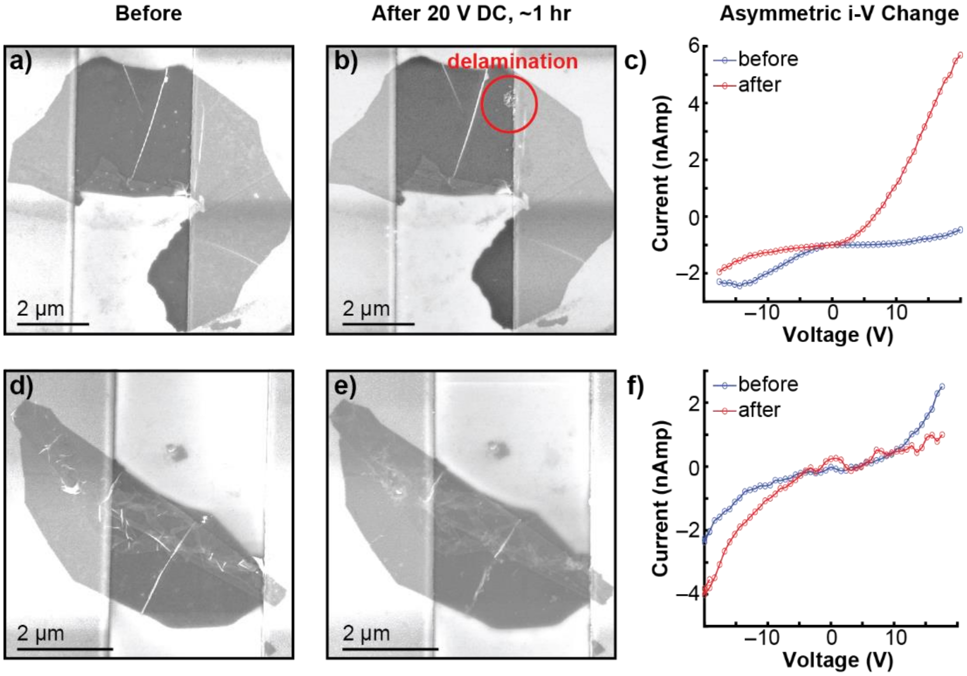

Figure S11 – SEM and I-V before and after 20 V DC stress. a,d) SEM images of a flake before and after (b,e) DC stress. One flake shows a trace consistent with delamination while the other shows no detectable morphology change. Before and after I-V traces for each flake are shown in (c,f).

These two flakes both exhibit asymmetric I-V changes after DC stress without showing signs of material thinning. Instead, one displays delamination, while the other shows no detectable morphological change by SEM. Together, they provide counterevidence to the notion that specific morphological changes directly correspond to specific I-V responses under DC stress.

XPEEM, AFM, SKPM

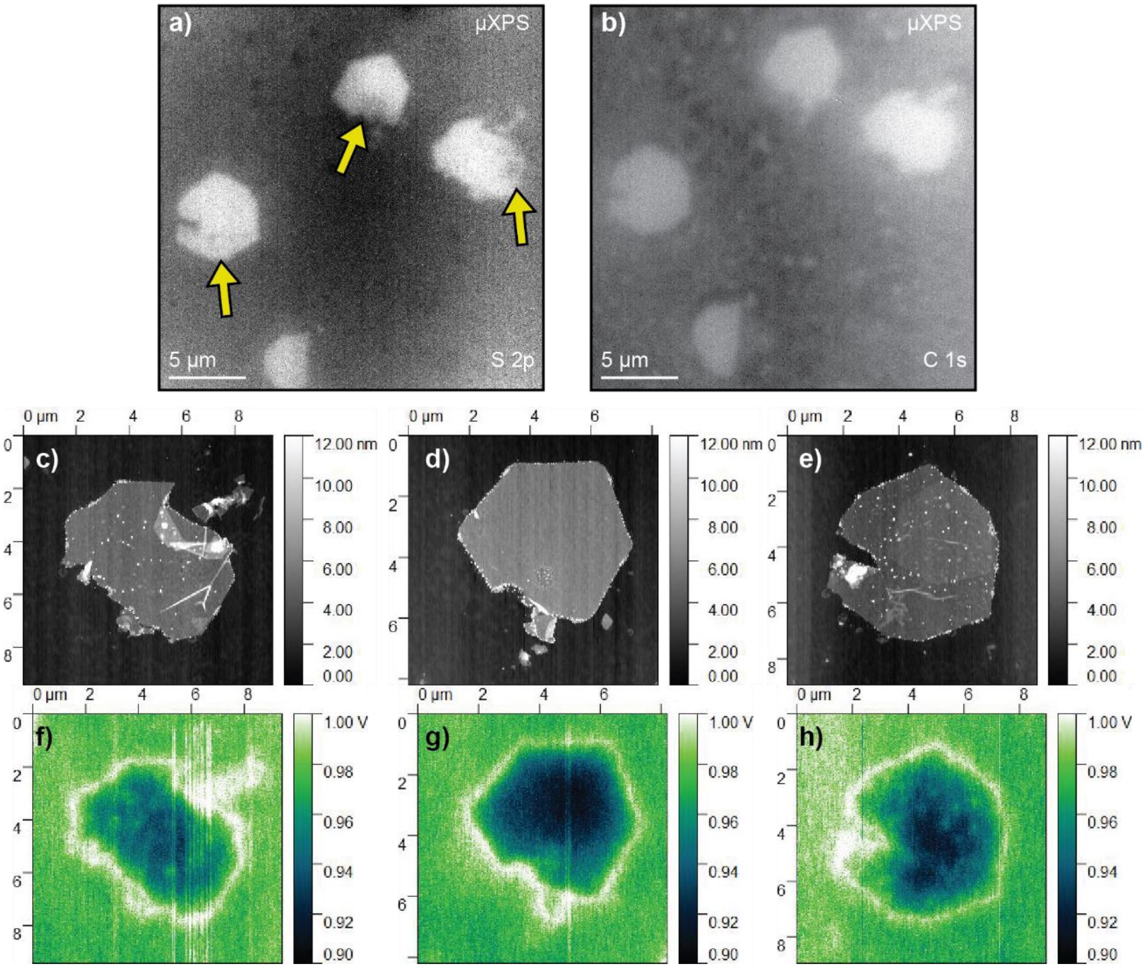

Figure S12 – XPEEM, AFM, and SKPM correlated data. a) µXPS of the S 2p peak. b) µXPS of the C 1s peak. c,d,e) Topography from AFM of three flakes indicated by yellow arrows in (a). f,g,h) corresponding flattened contact potential difference maps (CPD) from SKPM of the flakes corresponding to c,d,e respectively.

Low contrast features in the µXPS of S 2p do not correlate to any feature in the µXPS of C 1s; they are not due to C based surface contaminants from the transfer process. Further, there is no clear topological or contact surface potential (CPD) features that correlate to the low contrast features in the µXPS of S 2p.

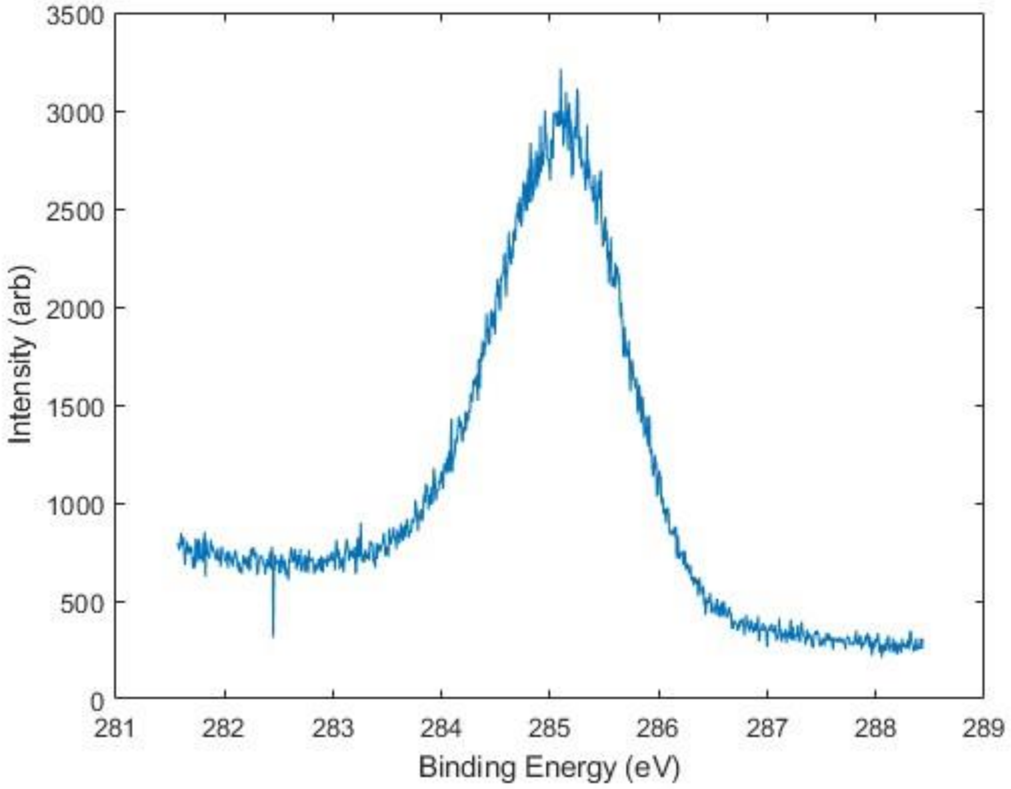

Figure S13 – XPS of C 1s taken with an x-ray photon energy of 350 eV.

Experimental setup

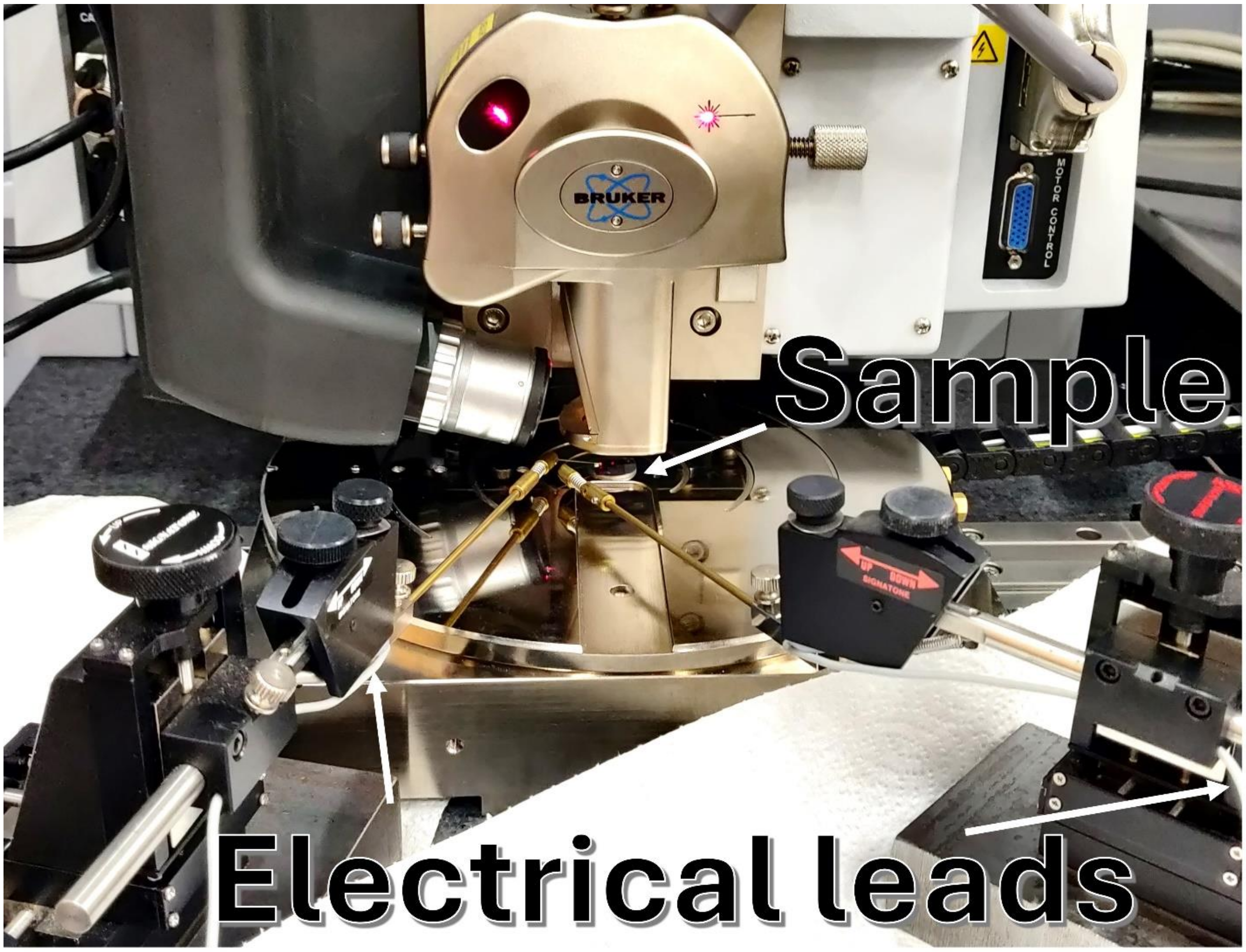

Figure S14 – Photograph of the experimental setup combing the Bruker Icon AFM head and electrical probes for simplified ex situ measurements.

Bibliography

(1) Sun, J.; Li, X.; Guo, W.; Zhao, M.; Fan, X.; Dong, Y.; Xu, C.; Deng, J.; Fu, Y. Synthesis Methods of Two-Dimensional MoS2: A Brief Review. *Crystals* **2017**, *7* (7), 198. https://doi.org/10.3390/cryst7070198.

(2) Nguyen, H.; Huang, C.-F.; Luo, W.; Xia, G. (Maggie); Chen, Z.; Li, Z.; Raymond, C.; Doyle, D.; Zhao, F. Synthesis of Large-Scale 2-D MoS2 Atomic Layers by Hydrogen-Free and Promoter-Free Chemical Vapor Deposition. *Mater. Lett.* **2016**, *168*, 1–4. https://doi.org/10.1016/j.matlet.2015.12.068.

(3) Zhang, W.; Zhang, P.; Su, Z.; Wei, G. Synthesis and Sensor Applications of MoS2-Based Nanocomposites. *Nanoscale* **2015**, *7* (44), 18364–18378. https://doi.org/10.1039/C5NR06121K.

(4) Bilgin, I.; Liu, F.; Vargas, A.; Winchester, A.; Man, M. K. L.; Upmanyu, M.; Dani, K. M.; Gupta, G.; Talapatra, S.; Mohite, A. D.; Kar, S. Chemical Vapor Deposition Synthesized Atomically Thin

Molybdenum Disulfide with Optoelectronic-Grade Crystalline Quality. *ACS Nano* **2015**, *9* (9), 8822–8832. https://doi.org/10.1021/acsnano.5b02019.

(5) Owiredu, P. Atomically Thin Molybdenum Disulfide Prepared via Chemical Vapor Deposition and Mechanical Exfoliation: Aging Studies and Reliability Testing. **2024**.

(6) Li, H.; Zhang, Q.; Yap, C. C. R.; Tay, B. K.; Edwin, T. H. T.; Olivier, A.; Baillargeat, D. From Bulk to Monolayer MoS_2 : Evolution of Raman Scattering. *Adv. Funct. Mater.* **2012**, *22* (7), 1385–1390. https://doi.org/10.1002/adfm.201102111.

(7) Gupta, S.; Johnston, A.; Khondaker, S. Correlated KPFM and TERS Imaging to Elucidate Defect-Induced Inhomogeneities in Oxygen Plasma Treated 2D MoS2 Nanosheets. *J. Appl. Phys.* **2022**, *131* (16), 164303. https://doi.org/10.1063/5.0088330.

(8) Splendiani, A.; Sun, L.; Zhang, Y.; Li, T.; Kim, J.; Chim, C.-Y.; Galli, G.; Wang, F. Emerging Photoluminescence in Monolayer MoS2. *Nano Lett.* **2010**, *10* (4), 1271–1275. https://doi.org/10.1021/nl903868w.

(9) Bauer, E. *Surface Microscopy with Low Energy Electrons*; Springer: New York, NY, 2014. https://doi.org/10.1007/978-1-4939-0935-3.

(10) Shakya, J.; Kumar, S.; Kanjilal, D.; Mohanty, T. Work Function Modulation of Molybdenum Disulfide Nanosheets by Introducing Systematic Lattice Strain. *Sci. Rep.* **2017**, *7* (1), 9576. https://doi.org/10.1038/s41598-017-09916-5.

(11) Zheng, X.; Calò, A.; Cao, T.; Liu, X.; Huang, Z.; Das, P. M.; Drndic, M.; Albisetti, E.; Lavini, F.; Li, T.-D.; Narang, V.; King, W. P.; Harrold, J. W.; Vittadello, M.; Aruta, C.; Shahrjerdi, D.; Riedo, E. Spatial Defects Nanoengineering for Bipolar Conductivity in MoS2. *Nat. Commun.* **2020**, *11* (1), 3463. https://doi.org/10.1038/s41467-020-17241-1.

(12) Precner, M.; Polaković, T.; Qiao, Q.; Trainer, D. J.; Putilov, A. V.; Di Giorgio, C.; Cone, I.; Zhu, Y.; Xi, X. X.; Iavarone, M.; Karapetrov, G. Evolution of Metastable Defects and Its Effect on the Electronic Properties of MoS2 Films. *Sci. Rep.* **2018**, *8* (1), 6724. https://doi.org/10.1038/s41598-018-24913-y.

(13) Cho, K.; Park, W.; Park, J.; Jeong, H.; Jang, J.; Kim, T.-Y.; Hong, W.-K.; Hong, S.; Lee, T. Electric Stress-Induced Threshold Voltage Instability of Multilayer MoS2 Field Effect Transistors. *ACS Nano* **2013**, *7* (9), 7751–7758. https://doi.org/10.1021/nn402348r.

(14) Guo, Y.; Wei, X.; Shu, J.; Liu, B.; Yin, J.; Guan, C.; Han, Y.; Gao, S.; Chen, Q. Charge Trapping at the MoS2-SiO2 Interface and Its Effects on the Characteristics of MoS2 Metal-Oxide-Semiconductor Field Effect Transistors. *Appl. Phys. Lett.* **2015**, *106* (10), 103109. https://doi.org/10.1063/1.4914968.

(15) Kang, J.; Liu, W.; Banerjee, K. High-Performance MoS2 Transistors with Low-Resistance Molybdenum Contacts. *Appl. Phys. Lett.* **2014**, *104* (9), 093106. https://doi.org/10.1063/1.4866340.

(16) Park, W.; Park, J.; Jang, J.; Lee, H.; Jeong, H.; Cho, K.; Hong, S.; Lee, T. Oxygen Environmental and Passivation Effects on Molybdenum Disulfide Field Effect Transistors. *Nanotechnology* **2013**, *24* (9), 095202. https://doi.org/10.1088/0957-4484/24/9/095202.

(17) Thiele, S.; Eliseyev, I. A.; Smirnov, A. N.; Jacobs, H. O.; Davydov, V. Y.; Schwierz, F.; Pezoldt, J. Electric Bias-Induced Edge Degradation of Few-Layer MoS2 Devices. *Mater. Today Proc.* **2022**, *53*, 281–284. https://doi.org/10.1016/j.matpr.2021.05.298.

(18) Omichi, K.; Ramos-Sanchez, G.; Rao, R.; Pierce, N.; Chen, G.; Balbuena, P. B.; Harutyunyan, A. R. Origin of Excess Irreversible Capacity in Lithium-Ion Batteries Based on Carbon Nanostructures. *J. Electrochem. Soc.* **2015**, *162* (10), A2106. https://doi.org/10.1149/2.0591510jes.

(19) Yuan, R.; Guo, Y.; Gurgan, I.; Siddique, N.; Li, Y.-S.; Jang, S.; Noh, G. A.; Kim, S. H. Raman Spectroscopy Analysis of Disordered and Amorphous Carbon Materials: A Review of Empirical Correlations. *Carbon* **2025**, *238*, 120214. https://doi.org/10.1016/j.carbon.2025.120214.

(20) Gołasa, K.; Grzeszczyk, M.; Bożek, R.; Leszczyński, P.; Wysmołek, A.; Potemski, M.; Babiński, A. Resonant Raman Scattering in MoS2—From Bulk to Monolayer. *Solid State Commun.* **2014**, *197*, 53–56. https://doi.org/10.1016/j.ssc.2014.08.009.

(21) Blanco, É.; Afanasiev, P.; Berhault, G.; Uzio, D.; Loridant, S. Resonance Raman Spectroscopy as a Probe of the Crystallite Size of MoS2 Nanoparticles. *Comptes Rendus Chim.* **2016**, *19* (10), 1310–1314. https://doi.org/10.1016/j.crci.2015.08.014.
(22) Tuschel, D. Resonance Raman and Photoluminescence Spectroscopy and Imaging of Few-Layer MoS2. **2015**, *30*.
